# Rain Evaporation, Snow Melt, and Entrainment at the Heart of Water Vapor Isotopic Variations in the Tropical Troposphere, According to Large‐Eddy Simulations and a Two‐Column Model

**DOI:** 10.1029/2020MS002381

**Published:** 2021-04-08

**Authors:** Camille Risi, Caroline Muller, Peter Blossey

**Affiliations:** ^1^ Laboratoire de Meteorologie Dynamique IPSL CNRS Ecole Normale Superieure Sorbonne Universite PSL Research University Paris France; ^2^ Department of Atmospheric Sciences University of Washington Seattle WA USA

**Keywords:** convection, large‐eddy simulation, water isotopes

## Abstract

We aim at developing a simple model as an interpretative framework for the water vapor isotopic variations in the tropical troposphere over the ocean. We use large‐eddy simulations of disorganized convection in radiative‐convective equilibrium to justify the underlying assumptions of this simple model, to constrain its input parameters and to evaluate its results. We also aim at interpreting the depletion of the water vapor isotopic composition in the lower and midtroposphere as precipitation increases, which is a salient feature in tropical oceanic observations. This feature constitutes a stringent test on the relevance of our interpretative framework. Previous studies, based on observations or on models with parameterized convection, have highlighted the roles of deep convective and mesoscale downdrafts, rain evaporation, rain‐vapor diffusive exchanges, and mixing processes. The interpretative framework that we develop, valid in case of disorganized convection, is a two‐column model representing the net ascent in clouds and the net descent in the environment. We show that the mechanisms for depleting the troposphere as the precipitation rate increases all stem from the higher tropospheric relative humidity. First, when the relative humidity is larger, less snow sublimates before melting and a smaller fraction of rain evaporates. Both effects lead to more depleted rain evaporation and eventually more depleted water vapor. This mechanism dominates in regimes of large‐scale ascent. Second, the entrainment of dry air into clouds reduces the vertical isotopic gradient and limits the depletion of tropospheric water vapor. This mechanism dominates in regimes of large‐scale descent.

## Key Points

- Isotopic enrichment of tropospheric water vapor by rain evaporation is stronger when drier air enhances sublimation and evaporation
- Entrainment of dry air weakens the vertical isotopic gradient and limits the depletion of tropospheric water vapor
- These mechanisms explain the increased depletion of tropospheric water vapor as tropospheric relative humidity increases

## 1 Introduction

### 1.1 Looking for an Interpretative Framework for Water Vapor Isotopic Profiles

The isotopic composition of water vapor (e.g., its Deuterium content, commonly expressed as $\delta D=\left(R/R_{SMOW}-1\right)\times 1000$ in ‰, where *R* is the ratio of Deuterium over Hydrogen atoms in the water, and SMOW is the Standard Mean Ocean Water reference) evolves along the water cycle as phase changes are associated with isotopic fractionation. Consequently, the isotopic composition of precipitation recorded in paleoclimate archives has significantly contributed to the reconstruction of past hydrological changes (Wang et al., 2001). It has also been suggested that observed isotopic composition of water vapor could help better understand atmospheric processes and evaluate their representation in climate models, in particular convective processes (Bony et al., 2008; Field et al., 2014; Lee et al., 2009; Schmidt et al., 2005). Yet, water isotopes remain rarely used beyond the isotopic community to answer today's pressing climate questions. A prerequisite to better assess the strengths and weaknesses of the isotopic tool is to better understand what controls spatiotemporal variations in water vapor isotopic composition (*δD*_*v*_) through the tropical troposphere, and in particular how convective processes drive these variations.

While there are interpretative frameworks for the controls of free tropospheric humidity (Romps, 2014; Sherwood, 1996), no such interpretative framework exist for water isotopes beyond the simple Rayleigh distillation or mixing lines (Bailey et al., 2017; Worden et al., 2007). We aim at filling this gap here. The first goal of this paper is thus to design an interpretative framework to interpret water vapor isotopic variations in the troposphere and to compare the processes controlling relative humidity and isotopic composition.

Frameworks do exist to interpret the *δD*_*v*_ in the subcloud layer (SCL), such as the (Merlivat & Jouzel, 1979) closure assumption, later extended to account for mixing with free tropospheric air (Benetti et al., 2015) and for updrafts and downdrafts (Risi et al., 2020). This latter framework highlighted the need to know the steepness of the relationship between *δD*_*v*_ and specific humidity *q* as they evolve with altitude. This motivates us to develop a framework that allows us to predict the *δD*_*v*_ evolution with altitude in the troposphere.

Here as a first step, we will focus on tropical oceans. This spares us the complications associated with land‐atmosphere interactions and topography and limits the effects of large‐scale horizontal advection that are so crucial at higher latitudes (Rozanski et al., 1993). At the same time, since water vapor over tropical oceans is a major source of water vapor and precipitation over many regions of the globe, understanding what controls *δD*_*v*_ over tropical oceans is a relevant and necessary first step.

### 1.2 Large‐Eddy Simulation Analysis as a Guide to Design the Interpretative Framework

Even in the most intensive field campaigns, the collected data remains scarce. For detailed process studies of convective processes, simulated three‐dimensional fields of meteorological variables are thus necessary (Randall, Krueger, et al., 2003). Many previous studies investigating the processes controlling tropospheric *δD*_*v*_ have relied on general circulation models that include convective parameterization (Bony et al., 2008; Field et al., 2010; Lee et al., 2007; Risi, Bony, & Vimeux, 2008). However, parameterizations include numerous simplifications or assumptions that are responsible for a significant part of biases in the present climate simulated by GCMs and of intermodel spread in climate change projections (Randall, Khairoutdinov, et al., 2003; Stevens & Bony, 2013; Webb et al., 2015). Therefore, here we use large‐eddy simulations (LES) with a resolution of 750 m, which are able to explicitly resolve convective motions.

The simulated three‐dimensional fields of meteorological and isotopic variables represent a huge amount of data that needs some interpretative framework to be interpreted. This is why many process studies based on LES develop an analytical or simple model to interpret LES results, e.g., Bretherton et al. (2005) and Romps (2011). Here we use the interpretative framework to quantify the relative contributions of different processes to the amount effect. The LES results serve as a guide to design the interpretative framework, provide its input parameters and serve as a benchmark to evaluate its results.

### 1.3 Interpreting the Amount Effect

Over tropical oceans, it has long been observed that in average over a month or longer, the isotopic composition of the rain is more depleted when the precipitation rate is stronger (Dansgaard, 1964; Rozanski et al., 1993). This is called the “amount effect.” Since most of the precipitation in the tropics is associated with deep convection, understanding the amount effect is a stringent test on our understanding of how convective processes affect the water isotopic composition in the tropical troposphere. The second goal of this study is thus to better understand the processes underlying the amount effect, using the interpretative framework.

To interpret the amount effect, in this study we will focus on the water vapor, for three reasons. First, the precipitation and water vapor isotopic composition are often observed to vary in concert (Kurita, 2013; Tremoy et al., 2014). Second (Dansgaard, 1964), interpreted the amount effect by the progressive depletion of the vapor by convective storms and by exchanges between the rain and the vapor. If this is the case, the amount effect crucially depends on the isotopic composition of the vapor. Third, from a column‐integrated water budget perspective, the isotopic composition of precipitation depends on the relative proportion of precipitation that originates from horizontal advection and from surface evaporation (Lee et al., 2007; Moore et al., 2014), the former being more depleted because it has already been processed in clouds. In this view as well, the amount effect crucially depends on the isotopic composition of the vapor.

**Figure 1:**
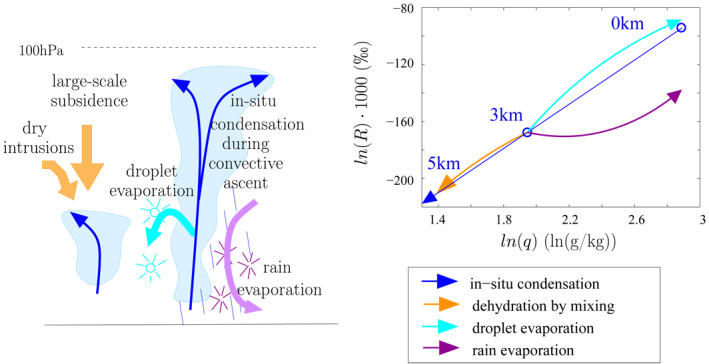
Schematic showing the influence of different processes on *q* and *δD*_*v*_. Condensation and immediate loss of condensate in convective updrafts leads to drying and depleting the water vapor following Rayleigh distillation (blue). During evaporation of cloud droplets, each droplet evaporates totally. Since cloud droplets are enriched in heavy isotopes, this moistens the air and enriches the vapor (cyan). In contrast, during evaporation of rain drops, each drop evaporates progressively. Whereas it moistens the air, it depletes the vapor for small evaporation fractions and enriches the vapor for large evaporation fraction (purple). Finally, mixing of dry air subsiding from higher altitudes (e.g., large‐scale subsidence), or transported from higher latitudes (e.g., dry intrusions) with air detrained from convective updrafts dehydrates the air and depletes the vapor following a hyperbolic curve (orange), leading to higher *δD*_*v*_ for a given *q* compared to Rayleigh. The curves are plotted following simple Rayleigh and mixing lines with approximate values taken from the control LES described later in the article. LES, large‐eddy simulations.

Water isotopic measurements in the vapor phase, by satellite or in situ, have confirmed that increased precipitation was associated with more depleted water vapor (Kurita, 2013; Lacour et al., 2017; Worden et al., 2007). Hereafter we will call this the “vapor amount effect.” In this paper, we will thus focus on understanding the processes underlying the “vapor amount effect.” Note that since precipitation and tropospheric humidity are generally related (Bretherton et al., 2004; Holloway & Neelin, 2009), the “vapor amount effect” can also be framed as the *δD*_*v*_ decrease as humidity increases (Lacour et al., 2017; Worden et al., 2007). From previous studies, four hypotheses have emerged:

1. Hypothesis 1: As precipitation rate increases, convective or mesoscale downdrafts bring more depleted vapor from above into the subcloud layer (SCL) (Kurita, 2013; Kurita et al., 2011; Risi, Bony, & Vimeux, 2008). This is because *δD*_*v*_ generally decreases with altitude, because as water vapor is lost through condensation, heavy isotopes are preferentially lost in the condensed phase following Rayleigh distillation (Figure 1, blue). However, downdrafts would both decrease *δD*_*v*_ and *q*. This hypothesis is thus inconsistent with the observation that *q* generally increases while *δD*_*v*_ decreases as precipitation rate increases.
2. Hypothesis 2: As precipitation rate increases, the moistening effect by rain evaporation increases. If the evaporated fraction of the rain is small, rain evaporation acts to deplete the vapor because light isotopes preferentially evaporate (Worden et al., 2007) (Figure 1, purple).
3. Hypothesis 3: As precipitation rate increases, the rain evaporation is more depleted, because the fraction of the rain that evaporates is smaller. As a larger fraction of the raindrop evaporates, the vapor produced by evaporation becomes less depleted and can sometimes be more enriched than the surrounding vapor (Risi, Bony, & Vimeux, 2008; Risi, Bony, Vimeux, Chong, & Descroix, 2010; Risi et al., 2020; Tremoy et al., 2014) (Figure 1, purple). In addition, larger precipitation rates typically occur in moister environments, which favor rain‐vapor diffusive exchanges rather than pure evaporation (Lawrence et al., 2004; Lee & Fung, 2008). Since rain comes from higher altitudes, it is more depleted than if in equilibrium with the local vapor, and thus rain‐vapor diffusive exchanges favor more depleted evaporation.
4. Hypothesis 4: As precipitation rate decreases, dehydration by mixing dominates relative to dehydration by condensation. Due to the hyperbolic shape of the mixing lines in a *q* − *δD* diagram, dehydration by mixing with a dry source is associated with a smaller depletion than predicted by Rayleigh distillation (Bailey et al., 2017; Dessler & Sherwood, 2003; Galewsky & Hurley, 2010; Galewsky & Rabanus, 2016) (Figure 1, orange).

The mechanisms underlying these hypotheses will have to be key ingredients of our interpretative framework. We notice that Hypotheses 2–4 are all associated with an increased steepness of the *q* − *δD*_*v*_ vertical gradients as precipitation rate increases (Figure 1), consistent with the key role of this steepness in depleting the SCL water vapor (Risi et al., 2020).

The LES will be described and analyzed in section 2. The interpretative framework will be designed and used to interpret the “vapor amount effect” in section 3. Finally, section 4 will offer a summary, some discussion and perspectives.

## 2 Large‐Eddy Simulations

### 2.1 Model and Simulations

We use the same LES model as in Risi et al. (2020), namely the System for Atmospheric Modeling (SAM) nonhydrostatic model (Khairoutdinov & Randall, 2003), version 6.10.9, which is enabled with water isotopes (Blossey et al., 2010). This model solves anelastic conservation equations for momentum, mass, energy, and water, which is present in the model under six phases: water vapor, cloud liquid, cloud ice, precipitating liquid, precipitating snow, and precipitating graupel. We use the bulk, mixed‐phase microphysical parameterization from Thompson et al. (2008) in which water isotopes were implemented (Moore et al., 2016).

The control simulation (“ctrl”) is three‐dimensional, with a doubly periodic domain of 96  × 96 km. The horizontal resolution is 750 m. There are 96 vertical levels. The simulation is run in radiative‐convective equilibrium over an ocean surface. The sea surface temperature (SST) is 30 °C. There is no rotation and no diurnal cycle. In this simulation, there is no large‐scale circulation.

The amount effect can be seen only if the precipitation increase is associated with a change in the large‐scale circulation (Bony et al., 2008; Dee et al., 2018; Risi et al., 2020). To compare ctrl to simulations with larger and smaller precipitation rate, we thus run simulations with a prescribed large‐scale vertical velocity profile, *ω*_*LS*_. This profile is used to compute large‐scale tendencies in temperature, humidity and water vapor isotopic composition. We compute large‐scale vertical advection by a simple upstream scheme (Godunov, 1959). In the computation, large‐scale horizontal gradients in temperature, humidity, and isotopic composition are neglected, i.e., there are no large‐scale horizontal advective forcing terms. The large‐scale vertical velocity *ω*_*LS*_ has a cubic shape so as to reach its maximum *ω*_*LSmax*_ at a pressure *p*_*max*_ = 500 hPa and to smoothly reach 0 at the surface and at 100 hPa (Bony et al., 2008). We analyze here simulations with *ω*_*LSmax*_ = 0 hPa/d (“ctrl”), corresponding to moderate deep‐convective conditions, *ω*_*LSmax*_ = −60 hPa/d (“HighPrec”), corresponding to typical deep‐convective conditions in the intertropical convergence zone, and *ω*_*LSmax*_ = +20 hPa/d (“LowPrec”), corresponding to subsiding trade‐wind conditions. The mean precipitation rates are 1.5, 2.5, and 8.5 mm/d, respectively, in LowPrec, ctrl, and HighPrec.

The simulations are run for 50 days. We use instantaneous outputs that are generated at the end of each simulation day. Only the last 10 days are analyzed, when the statistical radiative‐convective equilibrium is reached. In summary, the amount effect that is observed in reality across different seasons and locations (Dansgaard, 1964) or at the intraseasonal time scale (Kurita et al., 2011) is simulated here across different simulations, in average over the domain and over time.

In all our simulations, convection is disorganized, with isolated and short‐lived cumulonimbi (Figure S1). Therefore, our study does not represent organized states of convection. Since convective organization has been related to the amount effect in some studies (Kurita, 2013), aspects that may be altered by such organization will be specified along the paper.

### 2.2 Simulated Amount Effect and Evaluation with Respect to Observations

From LowPrec to ctrl and HighPrec, the domain‐mean precipitation increases, the air gets moister and the domain‐mean *δD* decreases both in the near‐surface vapor and in the precipitation, which vary in concert (Figures 3a and 3b, filled symbols). This is consistent with the amount effect.

**Figure 2:**
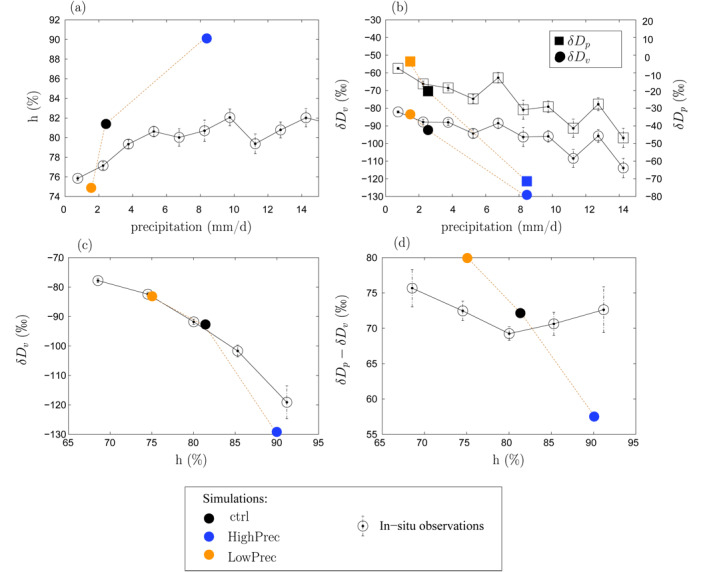
(a) Domain‐mean near‐surface relative humidity *h* as a function of domain‐mean precipitation rate, for the simulations (filled symbols) and observations (empty symbols with error bars). (b) Same as (a) but for near‐surface water vapor (circles) and precipitation (squares) *δD* as a function of domain‐mean precipitation rate. (c) Same as (a) but for the domain‐mean near‐surface *δD*_*v*_ as a function of the near‐surface (h) (d) Same as (a) but for the domain‐mean near‐surface *δD*_*p*_ − *δD*_*v*_ difference as a function of the near‐surface (h) In a and b, all observations are binned as a function of the TRMM precipitation rate in average over the surrounding 1.5° × 1.5° domain, with a bin width of 1.5 mm/d. In c and d, all observations are binned as a function of observed *h* with a bin width of 5%. The error bars indicate the standard deviation divided by the square root of the number of observations. Only *δD*_*p*_ observations that could be colocated with *δD*_*v*_ observations are considered.

**Figure 3:**
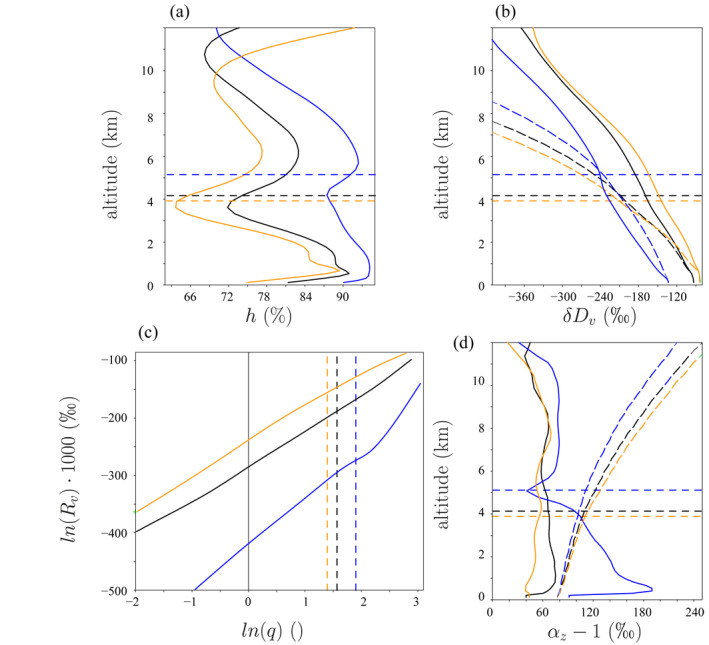
Vertical distribution of relative humidity (a), *δD*_*v*_ (b) and *α*_*z*_ (d) in ctrl (black), HighPrec (blue) and LowPrec (orange). (c) ln(*R*_*v*_(*z*)) ⋅ 1,000 as a function of ln(*q*(*z*)) for different altitudes. In b and d, dashed lines indicate the prediction by Rayleigh distillation. The horizontal lines in a, b, and d, and the vertical lines in c, show the altitude of the melting level. The dotted lines in a, b, and d show the altitude of the SCL top.

To assess the realism of this simulated amount effect, we compare our simulations to daily in situ isotopic observations both in the near‐surface vapor and in the precipitation collected during several cruises across the Pacific Ocean from 2006 to 2012 (Kurita, 2013). We colocate these observations with daily TRMM (Tropical Rainfall Measuring Mission) precipitation (Huffman et al., 2007) averaged over 1.5° × 1.5° domains, consistent with (Kurita, 2013). We average the precipitation over large domains for two reasons. First, as integrators of the water cycle, the isotopic composition of precipitation or water vapor is linked more tightly to precipitation when averaged over large scales (Gao et al., 2013; Risi, Bony, Vimeux, et al., 2008; Vimeux et al., 2005). Second, the doubly periodic domain in our simulations is meant to represent a large tropical area (e.g., in ctrl, large enough for radiative‐equilibrium to hold).

The simulations overestimate the sensitivity of near‐surface relative humidity *h*, *δD*_*v*_, and *δD*_*p*_ to domain‐mean precipitation amount (Figures 2a and 2b). Yet, the decrease of near‐surface *δD*_*v*_ as *h* increases is captured by our simulations with the correct order of magnitude (Figure 2c). This suggests that the overestimated sensitivities of *h*, *δD*_*v*_, and *δD*_*p*_ to domain‐mean precipitation amount all share the same reason. A first possible reason is the neglect of horizontal isotopic gradients (Bony et al., 2008; Risi, Bony, Vimeux, & Jouzel, 2010; Risi et al., 2019). Horizontal advection acts to bring enriched water vapor from dry to deep‐convective regions, damping daily *δD*_*v*_ variations in regions of large‐scale ascent by about 25% (Risi et al., 2019). A second possible reason is the disorganized state of convection in our simulations, with isolated, short‐lived cumulonimbi. In reality, convection is often organized and takes the form of mesoscale convective systems (Houze, 2004). Organized convection is drier than disorganized convection by up to 10% for a given large‐scale precipitation rate (Tobin et al., 2012), which may explain the drier observations. It may similarly explain the more enriched observations. Although investigating the impact of convective organization on the amount effect is beyond the scope of this paper, we will keep in mind that the lack of convective organization in our simulations is a caveat of our study.

In spite of the overestimated sensitivity of *h* and *δD*_*v*_ to precipitation rate, the simulations correctly simulate the sensitivity of *δD*_*v*_ to *h*. In other words, they correctly simulate the “vapor amount effect” when framed as the *δD*_*v*_ decrease as humidity increases (Lacour et al., 2017; Worden et al., 2007). In this paper, the relative humidity variations will be shown to be essential to the “vapor amount effect.” The correct simulation of the relationship between *δD*_*v*_ and relative humidity gives confidence in the simulated mechanisms underlying this relationship.

Finally, the order of magnitude of the *δD*_*p*_ − *δD*_*v*_ difference is also well captured (Figure 2d). This gives confidence that the rain‐vapor exchanges, which we will demonstrate to be key to the “vapor amount effect,” are properly represented.

### 2.3 Humidity and *δD*_*v*_ Vertical Profiles and Steepness of the *q* − *δD*_*v*_ Relationship

In HighPrec, the domain‐mean relative humidity *h* is larger than in ctrl by more than 10% (Figure 3b), mainly due to the moistening by large‐scale ascent (Section 3.2.1), while *δD*_*v*_ is more depleted by more than 50‰, in most of the troposphere (Figure 3c). We can see that the *δD*_*v*_ difference at all altitudes is similar to that in the SCL (here we define the SCL as the highest level where the domain‐mean condensation rate remains below 10^−1^ g/kg/d). This is because the SCL ultimately feeds the water vapor at all altitudes in the troposphere. This confirms that understanding what controls the SCL *δD*_*v*_ is key to understand what controls *δD*_*v*_ at all altitudes (Risi et al., 2020). This also explains why models that assume constant SCL *δD*_*v*_ show very little sensitivity to all kinds of convective and microphysical processes (Duan et al., 2018). We can also see that Rayleigh distillation alone (dashed line) is a poor predictor of *δD*_*v*_ profiles and of their sensitivity to large‐scale circulation.

With the goal of understanding the “vapor amount effect,” as a first step (Risi et al., 2020) focused on understanding what controls the *δD*_*v*_ in the SCL. They identified the key role of the steepness of the *q* − *δD*_*v*_ relationship of vertical profiles in the lower troposphere, which determines the efficiency with which updrafts and downdrafts deplete the SCL. To understand what controls *δD*_*v*_ in the SCL and thus everywhere in the troposphere, we thus need to understand what controls the steepness of the *q* − *δD*_*v*_ relationship.

The vertical profiles of ln(*R*_*v*_) as a function of ln(*q*) for each simulation show a nearly linear relationship (Figure 3d), consistent with a Rayleigh‐like distillation process (Figure 1). If the vertical profiles were dominated by mixing processes, as in Hypothesis 4, the relationship would look concave down (Bailey et al., 2017) (Figure 1, orange). Rather, in HighPrec, the curve looks concave up near the melting level, consistent with an effect of rain evaporation (Figure 1, purple).

To better quantify the steepness of the *q* − *δD*_*v*_ relationship, we define the *q* − *δD*_*v*_ steepness *α*_*z*_, as the effective fractionation coefficient that would be needed in a distillation to fit the simulated joint *q* − *δD*_*v*_ evolution (Risi et al., 2020):

(1) $$\alpha_{z}=1+\frac{ln\left(R_{v}\left(z\right)/R_{v}\left(z-dz\right)\right)}{ln\left(q\left(z\right)/q\left(z-dz\right)\right)}$$

The steepness *α*_*z*_ in the ctrl simulation is smaller than that predicted by Rayleigh distillation, i.e., *α*_*z*_ < *α*_*eq*_, especially at higher altitudes (Figure 3e) (Section 3.2.2 will demonstrate that it is due to entrainment). Just above the SCL top, *α*_*z*_ − 1 is more than three times larger in HighPrec than in ctrl. The increased steepness leads the updrafts and downdrafts to deplete more efficiently the SCL water vapor (Risi et al., 2020), and eventually the full tropospheric profile through mixing by deep convection. Conversely, in LowPrec, the steepness is smaller and responsible for more enriched SCL. Our interpretative framework will allow us to interpret these features (Section 3).

### 2.4 Effect of Deactivating Rain‐Vapor Exchanges

According to Hypotheses 2 and 3, the isotopic composition of the rain plays a key role in the “vapor amount effect.” At a given instant and for a small increment of rain evaporation fraction, the isotopic composition of the evaporation flux *R*_*ev*_ is simulated following (Craig & Gordon, 1965):

$$R_{ev}=\frac{R_{r}/\alpha_{eq}-h_{ev}\cdot R_{v}}{\alpha_{K}\cdot \left(1-h_{ev}\right)}$$

where *R*_*r*_ and *R*_*v*_ are the isotopic ratios in the liquid water and water vapor, *α*_*eq*_ and *α*_*K*_ are the equilibrium and kinetic fractionation coefficient and *h*_*ev*_ is the relative humidity. In order to test Hypotheses 2 and 3, we run additional simulations similar to ctrl and HighPrec but without any fractionation during rain evaporation, named “nofrac,” where *R*_*ev*_ = *R*_*r*_. We also run additional simulations with fractionation during evaporation, but with rain‐vapor diffusive exchanges deactivated, named “nodiff,” where *R*_*ev*_ = *R*_*r*_/*α*_*eq*_/*α*_*K*_.

**Figure 4:**
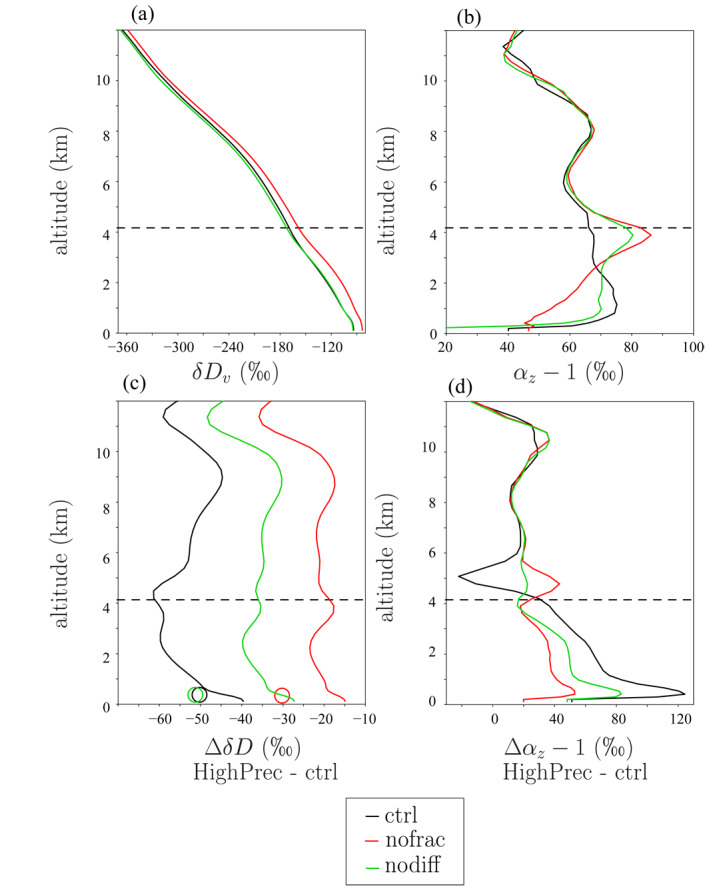
(a) Vertical distribution of *δD*_*v*_ for ctrl, when fractionation during liquid evaporation is turned on (black) or off (red) and when liquid‐vapor equilibration is turned off (green). (b) Same as (a) for the vertical profiles of *α*_*z*_. (c) *δD*_*v*_ difference between the HighPrec and ctrl, with (black) and without (red) fractionation during evaporation and when liquid‐vapor equilibration is turned off (green). The circles illustrate the difference in the precipitation *δD*.(d) Same as (c) but for *α*_*z*_.

When fractionation during rain evaporation is deactivated, *δD*_*v*_ is more enriched, consistent with a more enriched composition of rain evaporation (Figure 4a). In addition, the *δD*_*v*_ difference between HighPrec and ctrl is reduced by about 70% compared to when all isotopic exchanges are considered (Figure 4c, red). This confirms that fractionation during rain evaporation plays a key role in the “vapor amount effect.” When rain‐vapor diffusive exchanges are deactivated, the *δD*_*v*_ difference between HighPrec and ctrl is reduced by about 30% compared to when all isotopic exchanges are considered (Figure 4c, green). Rain‐vapor diffusive exchanges thus play an important role as well.

We note that the *δD*_*v*_ difference between the simulations is remarkably uniform with altitude (Figures 4a and 4c), although we expect strong vertical variations in rain evaporation. This is consistent with the important role of the SCL *δD*_*v*_ as an initial condition for the full *δD*_*v*_ profile. We also note that more enriched *δD*_*v*_ profiles are associated with a reduced lower‐tropospheric steepness *α*_*z*_ just above the SCL, and larger *δD*_*v*_ differences between simulations are associated with larger differences in lower‐tropospheric *α*_*z*_. This is consistent with the SCL *δD*_*v*_ being mainly driven by the steepness *α*_*z*_ just above the SCL (Risi et al., 2020). Finally, the reduced “vapor amount effect” in “nofrac” leads to a reduced amount effect in the precipitation *δD* as well (Figure 4c, circles). This shows that the column‐integrated water budget (Lee et al., 2007; Moore et al., 2014) cannot by itself predict the amount effect, since it depends on the isotopic composition of the advected vapor, which can greatly vary depending on the detailed representation of rain evaporation processes.

To summarize, in the total *δD*_*v*_ difference between HighPrec and ctrl, there is about one third due to fractionation during evaporation, one third due to rain‐vapor diffusive exchanges, and one third that would remain even in absence of any fractionation during evaporation. These tests suggest that Hypotheses 2 and/or 3 play a key role in the “vapor amount effect.” In the next sections, we aim at better understanding how rain evaporation impacts *δD*_*v*_ profiles.

### 2.5 Vertical Profiles Binned by Moist Static Energy

Previous studies have shown that analyzing variables in isentropic coordinates was a powerful tool to categorize the different convective structures: undiluted updrafts, diluted updrafts, saturated and unsaturated downdrafts, and the environment (Kuang & Bretherton, 2006; Pauluis & Mrowiec, 2013). This method also has the advantage of filtering out gravity waves. It has been applied to the analysis of a wide range of convective systems (Chen et al., 2018; Dauhut et al., 2017; Mrowiec et al., 2015, 2016).

Here we use the frozen moist static energy *m* as a conserved variable because it is conserved during condensation and evaporation of both liquid and ice water (Hohenegger & Bretherton, 2011; Muller & Romps, 2018).

$$m=c_{pd}\cdot T+g\cdot z+L_{v}\cdot q_{v}-L_{f}\cdot q_{i}$$

where *c*_*pd*_ is the specific heat of dry air, *T* is temperature, *g* is gravity, *z* is altitude, *L*_*v*_ and *L*_*f*_ are the latent heat of vapourization and fusion, and *q*_*i*_ is the total ice water content (cloud ice, graupel, and snow). At each level, we categorize all grid points into bins of *m* with a width of 0.4 kJ/kg.

**Figure 5:**
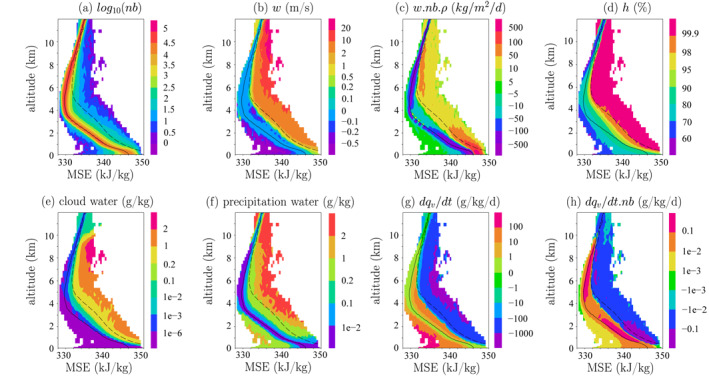
Variables binned as a function of frozen moist static energy *m* and of altitude, for the ctrl simulation: (a) number of samples, (b) vertical velocity anomaly, (c) vertical mass flux (vertical velocity multiplied by the proportion of samples and density), (d) relative humidity, (e) cloud water content mixing ratio (liquid and ice), (f) precipitating water mixing ratio (rain, graupel and snow), (g) evaporation and condensation tendency *dq*/*dt* (positive in case of evaporation, negative in case of condensation), (h) *dq*/*dt* multiplied by the number of samples. The solid black line shows the domain‐mean frozen moist static energy, while the dashed black line shows the frozen moist static energy at saturation.

The domain‐mean *m* decreases from the upper troposphere down to about 5 km, due to the loss of energy by radiative cooling, and then increases down to the surface due to the input of energy by surface fluxes (Figure 5, solid black line). Based on this diagram, we can identify four kinds of air parcels:

1. *Environment*. They correspond to air parcels whose *m* is close to the domain‐mean (solid black). They are the most numerous (Figure 5a). Their vertical velocity is slightly descending (Figure 5b), but because they are very numerous, they account for most of the downward mass flux (Figure 5c). Their relative humidity is close to the domain‐mean (Figure 5d), they contain only a small cloud water and rain content and phase changes are very slow (Figures 5e–5g). However, because they cover most of the domain, they contribute significantly to the evaporation in the domain‐mean (Figure 5h)
2. *Cloudy Updrafts*. They correspond to air parcels with *m* larger than the domain‐mean and whose bin‐mean vertical velocity is ascending (Figure 5b). If air rose adiabatically from the SCL, they would conserve their *m* and they would be located completely on the right of the diagram. In practice, *m* decreases because the environment air is progressively entrained into ascending parcels. In the diagrams, parcels are more diluted when they are closer to the domain‐mean, and less diluted when they are more to the right. In spite of their dilution with the environment, their humidity is at saturation (Figure 5d). They contain a lot of cloud and precipitating water, and vapor undergoes condensation (Figures 5e–5g)
3. *Cloudy Downdrafts*. They correspond to air parcels with *m* larger than the domain‐mean but whose bin‐mean vertical velocity is descending (Figure 5b). They are more diluted than cloudy updrafts. Their humidity is below saturation (Figure 5d). They contain cloud and precipitating water that undergo evaporation (Figures 5e–5g). Located around the cloudy updrafts in the real space, they mainly correspond to subsiding shells (e.g., Glenn & Krueger, 2014)
4. *Precipitating Downdrafts*. They correspond to air parcels below the melting level and with *m* lower than the domain‐mean. They are among the most strongly descending air parcels (Figure 5b) but since they are scarce (Figure 5b), contribute little to the total descending mass flux (Figure 5c). They are very dry, with no cloud water, but with precipitating water (Figures 5d–5f). We interpret these parcels as unsaturated, precipitating downdrafts. Strong evaporation of rain occurs in these downdrafts (Figure 5g), but because they cover only a small fraction of the domain, they contribute little to the evaporation in the domain‐mean (Figure 5h)

**Figure 6:**
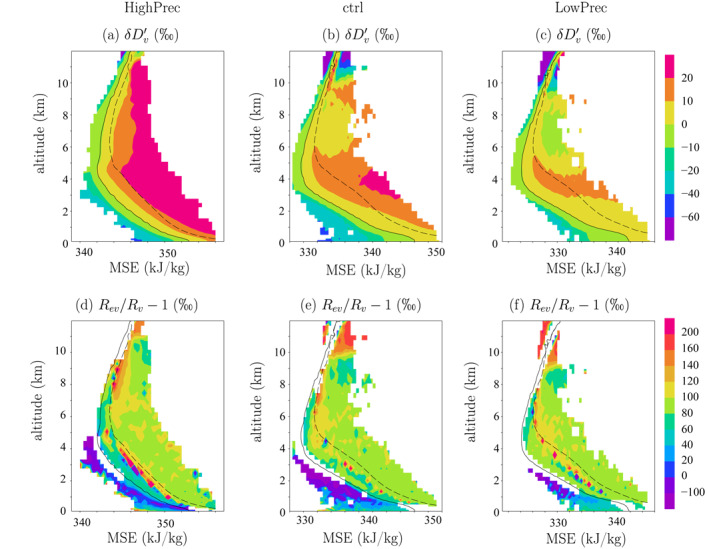
(b,e) As for Figure 5 but for (a) *δD*_*v*_ anomaly with respect to the domain‐mean at each vertical level, (d) (*ϕ* − 1) ⋅ 1,000, where *ϕ* = *R*_*ev*_/*R*_*v*_; it is expressed in ‰. (a,d) As for (b,e) but for HighPrec. (c,f) As for (b,e) but for LowPrec.

The isotopic composition of water vapor is most enriched in the least diluted updrafts, and most depleted in the precipitating downdrafts (Figure 6b). To assess the effect of phase changes, in each altitude and for each bin of *m*, we plot *ϕ* = *R*_*ev*_/*R*_*v*_, where *R*_*ev*_ is the ratio of the water vapor tendency associated with phase changes (evaporation in downdrafts and in the environment, or condensation in cloudy updrafts) and *R*_*v*_ is the isotopic ratio of the water vapor. In cloudy updrafts, *ϕ* − 1 is about 100‰ in the lower troposphere and increases with height (Figure 6e). This roughly corresponds to equilibrium fractionation during condensation. In cloudy downdrafts, *ϕ* − 1 is also about 100‰. This means that cloud droplets evaporate totally without fractionation. In contrast, in precipitating downdrafts, *ϕ* − 1 is much lower. It is around 30‰ below 1 km. The fact that *ϕ* − 1 is positive is consistent with the fact that rain evaporation in the SCL acts to slightly enrich the water vapor (Risi et al., 2020). In contrast, between 2 and 3 km, *ϕ* − 1 is around −100‰: at these levels, rain evaporation acts to deplete the water vapor, consistent with (Worden et al., 2007).

These diagrams look qualitatively similar for the other simulations. One noticeable difference is that in HighPrec, the *δD*_*v*_ contrast between the environment and the cloudy regions is larger (Figure 6a). This may be associated with the more depleted evaporation of the rain in precipitating downdrafts and of cloud droplets in cloudy downdrafts (Figure 6d). Conversely in LowPrec, the *δD*_*v*_ contrast between the environment and the cloudy regions is larger (Figure 6c). To quantitatively compare the different simulations, now we plot vertical profiles of variables in average over cloudy regions and over the environment.

### 2.6 Vertical Profiles for Cloudy Regions and for the Environment

Here we chose to define cloudy regions as all parcels with a cloud (liquid or ice) water content greater than 10^−6^ g/kg (e.g., Thayer‐Calder & Randall, 2015). In this loose definition, “cloudy regions” correspond to both cloudy updrafts and downdrafts, while the “environment” includes both the environment and precipitating downdrafts. Including the cloudy downdrafts into the cloudy regions is justified by the fact that a significant portion of the water condensed in cloudy updrafts subsequently evaporate in these cloudy downdrafts, without directly affecting the environment. Our results below are not crucially sensitive to the definition of the cloudy regions and of the environment, provided that the definition of cloudy regions is not too restrictive (Text S1).

**Figure 7:**
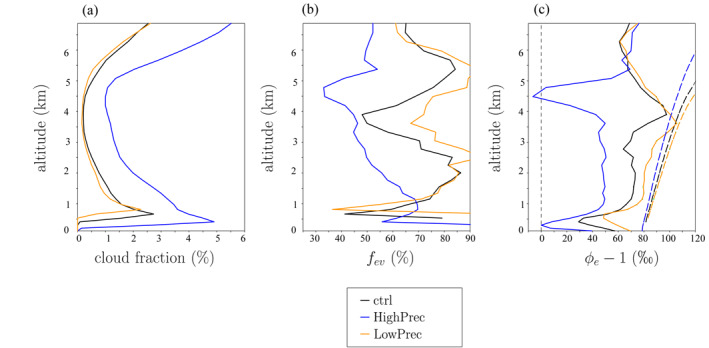
(a) Fraction of the domain covered by cloudy regions. (b) Fraction of the water condensed in cloudy regions that evaporates into the environment, *f*_*ev*_. (c) $\left(\phi -1\right)\cdot 1,000$ (solid) and $\left(\alpha_{eq}-1\right)\cdot 1,000$ (dashed), where *ϕ* = *R*_*ev*_/*R*_*e*_ and *α*_*eq*_ is the equilibrium fractionation coefficient. Both are expressed in ‰. The black, red, and green lines are for ctr, HighPrec and LowPrec, respectively.

Cloudy regions cover only a few percent of the domain (Figure 7a). The fraction of water condensed in cloudy regions that evaporates into the environment, estimated as *f*_*ev*_ = −(*dq*/*dt*)_*env*_/(*dq*/*dt*)_*cloud*_, where (*dq*/*dt*)_*env*_ and (*dq*/*dt*)_*cloud*_ are the humidity tendencies associated with phase changes in average in the environment and in the cloudy region, respectively, varies between 30% and 90%, depending on altitude (Figure 7b). It is smaller in HighPrec and than in ctrl, because the environment is moister.

Figure 7c plots *ϕ*_*e*_ = *R*_*ev*_/*R*_*e*_, where $R_{ev}={\left(dq_{HDO}/dt\right)}_{env}/{\left(dq/dt\right)}_{env}$, ${\left(dq_{HDO}/dt\right)}_{env}$ is the *HDO* tendency associated with phase changes in the environment and *R*_*e*_ is the isotopic ratio in the environment. In all simulations except in HighPrec near 4.5 km, *ϕ*_*e*_ > 1: the evaporation has an enriching effect on the environment. The overall enriching effect of evaporation contradicts Hypothesis 2. Yet in all cases, *ϕ*_*e*_ < *α*_*eq*_: the evaporation is not as enriching as if there was total evaporation of condensate. The *ϕ*_*e*_ is smaller in HighPrec and larger in LowPrec than in ctrl: rain evaporation has a weaker enriching effect in HighPrec and a stronger enriching effect in LowPrec. This supports Hypothesis 3. In HighPrec near 4.5 km, near the melting level, there is even a small layer where *ϕ*_*e*_ < 1: at this level, the rain evaporation has a depleting effect on the water vapor.

### 2.7 What Controls the Isotopic Composition of Rain Evaporation?

Why is *ϕ* smaller in HighPrec and higher in LowPrec than in ctrl? It could be because rain‐vapor exchanges in a moister environment leads the evaporation to have a more depleting effect (Lawrence et al., 2004; Risi, Bony, & Vimeux, 2008), or because rain evaporation is more depleted when the evaporated fraction is small (Risi, Bony, & Vimeux, 2008; Tremoy et al., 2014), or because the rain itself is more depleted. We aim here at quantifying these different effects.

**Figure 8:**
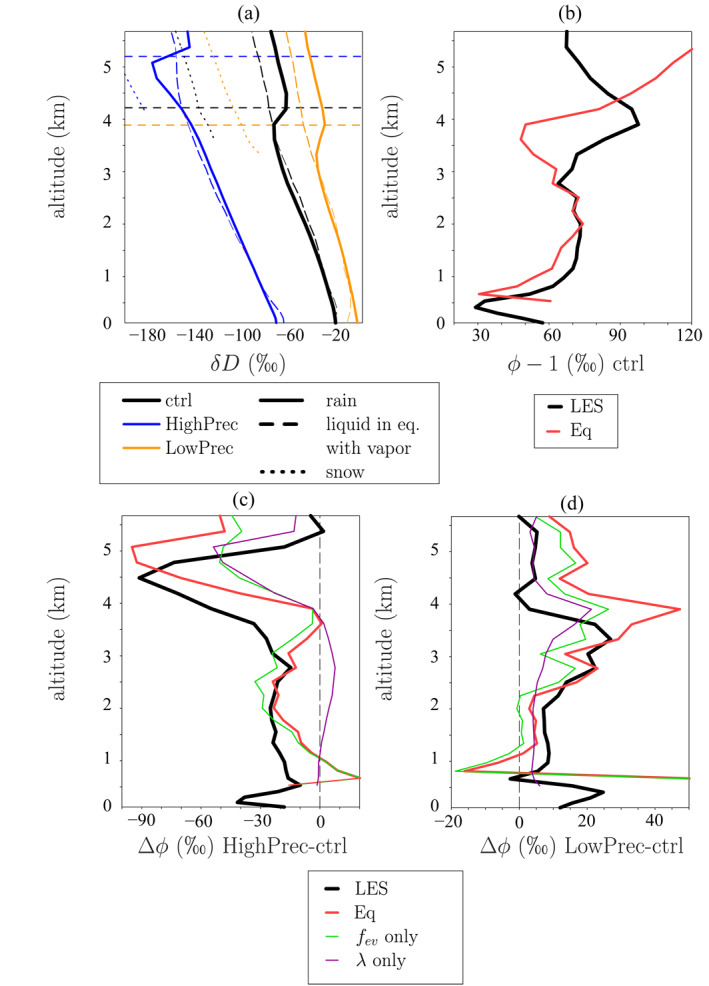
(a) *δD* profile for rain water (solid) and snow (dotted) falling in the environment. The liquid that would be in equilibrium with the vapor in the environment is shown in dashed. (b) Profile of *ϕ*_*e*_ = *R*_*ev*_/*R*_*e*_ simulated by the ctrl simulation (black, same as in Figure 7c, black) and predicted by Equation 2 (red). (c) Difference of *ϕ* between HighPrec and ctrl simulated by the LES (black), predicted by equation 2 (red), predicted by Equation (2) if only *f*_*ev*_ varies (green) and if only *λ* varies (purple). (d) Same as (c) but for the difference between LowPrec and ctrl.

Figure 8a plots the vertical profiles of rain *δD* (solid). Below the melting level, the rain is very close to isotopic equilibrium with the vapor (dashed). Above the melting level, the rain is more enriched than if in equilibrium due to rain lofting. Near the melting level for simulation HighPrec, the rain is anomalously depleted. This is due to snow melt. Since the snow forms higher in altitude, it is more depleted than the rain. It thus imprints its depleted signature on the rain when melting. In HighPrec, the moist middle troposphere prevents most of the snow from sublimating: 24% of the precipitation is made of snow at the melting level. The rain is thus strongly depleted by snow melt. In contrast, in ctrl and LowPrec, the drier middle troposphere favors snow sublimation: only 8% and 3% of the precipitation is made of snow at the melting level, respectively.

The quick equilibration between the rain and vapor motivates us to use a simple equation in which some mass *q*_*l*0_ of rain, with isotopic ratio *R*_*l*0_, partially evaporates and isotopically equilibrates with some mass *q*_*e*0_ of environment vapor, with isotopic ratio *R*_*e*0_. As explained in Text S2, if *q*_*l*0_ ≫ *q*_*e*0_, we get:

(2) $$\phi_{e}=\frac{\lambda }{1+\left(1-f_{ev}\right)\cdot \left(\alpha_{eq}-1\right)}$$

where *ϕ*_*e*_ = *R*_*ev*_/*R*_*e*_, *λ* = *R*_*l*0_/*R*_*e*0_, *R*_*ev*_ is the isotopic ratio of the rain evaporation flux, *α*_*eq*_ is the equilibrium fractionation coefficient and *f*_*ev*_ is the fraction of the rain that evaporates. Equation (2) tells us that the rain evaporation is more depleted as the rain is more depleted relative to the vapor (quantified by *λ*) and as the evaporated fraction *f*_*ev*_ is smaller. This simple equation (Figure 8b, red) is able to approximate the simulated values of *ϕ*_*e*_ (black) for the ctrl simulation and is able to capture the smaller and larger values of *ϕ*_*e*_ for HighPrec and LowPrec, respectively (Figures 8c and 8d).

We find that below the melting level, *ϕ*_*e*_ is smaller in HighPrec than in ctrl mainly because *f*_*ev*_ is smaller (Figure 8c, green). Near the melting level, *ϕ* is smaller in HighPrec than in ctrl both because *f*_*ev*_ is smaller and because *λ* is smaller, i.e., the rain is more depleted due to snow melt (Figure 8c, purple). In LowPrec, the effect of *f*_*ev*_ dominates at most levels (Figure 8d).

## 3 Summary

To summarize, the previous sections suggest that rain evaporation in the lower troposphere is a key ingredient of the “vapor amount effect.” The isotopic composition of the rain evaporation flux mainly depends on the evaporated fraction of the rain, consistent with (Risi, Bony, & Vimeux, 2008; Tremoy et al., 2014). Near the melting level in regimes of large‐scale ascent, it is also impacted by snow melt. We hypothesize that the isotopic effect of rain evaporation propagates downward down to the SCL. To test this hypothesis and to understand the underlying mechanisms, in the next section we develop a simple two‐column model.

## 4 A Simple Two‐Column Model to Quantify the Relative Contributions of Different Processes

The previous section and previous studies provide a guide for developing our simple interpretative framework. First, the model needs to represent the effect of rain evaporation, highlighted as a key process in the previous section. Second, alternative hypotheses for the “vapor amount effect” involve mixing between the subsiding environment and detrained water (Bailey et al., 2017) (Hypothesis 4). This process also needs to be represented in our model. Third, the steepness of the *q* − *δD*_*v*_ relationship must be a key ingredient, since it drives *δD*_*v*_ in the SCL and thus *δD*_*v*_ everywhere. Finally, the previous section has relied on the distinction between the environment and cloudy regions. Keeping this distinction, we develop a two‐column model.

### 4.1 Model Equations and Numerical Application to LES Outputs

#### 4.1.1 Balance Equations

**Figure 9:**
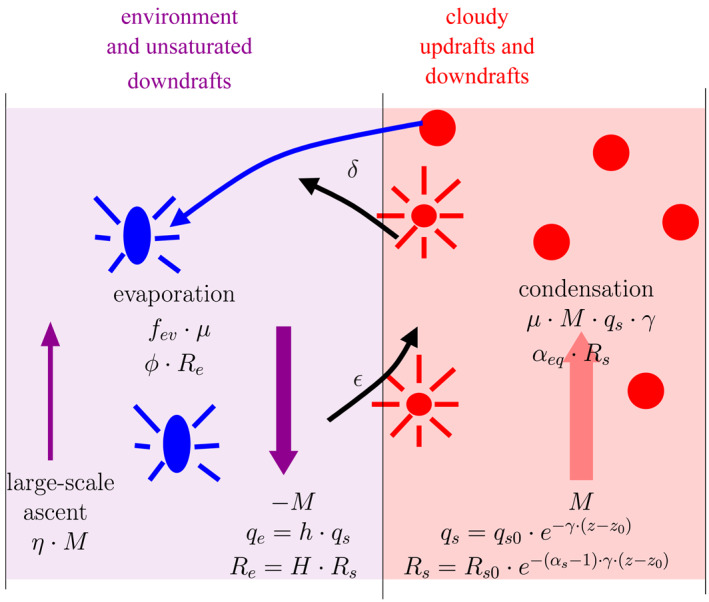
Schematic view of the simple two‐column model, and definition of the main variables. In the cloudy region, air with specific humidity *q*_*s*_ and isotopic ratio *R*_*s*_ ascends with mass flux *M*, entrains air with entrainment rate *ϵ*, condenses with efficiency *μ*, and detrains with entrainment rate *δ*. In the environment, air with specific humidity *q*_*e*_ and isotopic ratio *R*_*e*_ descends with mass flux *M*, is entrained into the cloudy region and is progressively remoistened by detrainment from cloudy regions, rain evaporation with evaporated fraction *f*_*ev*_ and by the large‐scale vertical advection tendency equivalent to an ascending mass flux *η* ⋅ *M*.

This model is inspired by the two‐column model used to predict tropospheric relative humidity in Romps (2014) and *δD*_*v*_ profiles in Duan et al. (2018). The first column represents the cloudy regions, including cloudy updrafts and downdrafts, as a bulk entraining plume. The second column represents the subsiding environment and precipitating downdrafts (Figure 9).

The mass balance for the air in the cloudy regions writes:

(3) $$\frac{dM}{dz}=M\cdot \left(\epsilon -\delta \right)$$

where *M* is the bulk mass flux in the cloudy regions (positive upward), *ϵ* and *δ* are the fractional entrainment and detrainment rates.

We assume that the *q* in the cloudy regions is at saturation, and call it *q*_*s*_. The water balance in the cloudy regions writes:

(4) $$\frac{d\left(Mq_{s}\right)}{dz}=\epsilon \cdot M\cdot q_{e}-\delta \cdot M\cdot q_{s}-c$$

where *c* is the condensation rate and *q*_*e*_ is the specific humidity in the environment. The terms on the right‐hand side represent the water input by entrainment of environment air, the water loss by detrainment of cloudy air, and the water loss by condensation, respectively. We assume that all the condensed water is immediately lost by the cloudy regions to the environment, and evaporation of this lost water can occur in the subsaturated environment only, as in Romps (2014).

We assume that mass is conserved within the domain, so that the flux in the environment is − *M*. The large‐scale ascent, when present, is taken into account through a humidity tendency, consistent with the LES set‐up. We assume that the large‐scale humidity tendency applies to the environment only, which is a first‐order approximation justified by the small fraction of the domain that is covered by cloudy updrafts (less than 10%). The water balance in the environment writes:

(5) $$\frac{d\left(-Mq_{e}\right)}{dz}=-\epsilon \cdot M\cdot q_{e}+\delta \cdot M\cdot q_{s}+f_{ev}\cdot c-\eta \cdot M\cdot \frac{\partial q_{e}}{\partial z}$$

where *f*_*ev*_ is the fraction of the cloud or precipitating water that evaporates in the environment, *η* = *M*_*LS*_/*M* and *M*_*LS*_ is the domain‐mean large‐scale mass flux. The terms on the right‐hand side represents the water loss by entrainment into cloudy regions, water input by the detrainment of cloudy air, partial evaporation of condensed water, and water input by large‐scale vertical advection.

Regarding water isotopes, we assume that the cloud water removed by condensation is in isotopic equilibrium with the cloudy region water vapor. The isotopic balance in the cloudy regions thus writes:

(6) $$\frac{d\left(Mq_{s}\cdot R_{s}\right)}{dz}=\epsilon \cdot M\cdot q_{e}\cdot R_{e}-\delta \cdot M\cdot q_{s}\cdot R_{s}-c\cdot \alpha_{eq}\cdot R_{s}$$

where *α*_*eq*_ is the equilibrium fractionation coefficient, *R*_*s*_ is the isotopic ratio in the cloudy regions, and *R*_*e*_ is the isotopic ratio in the environment.

The isotopic balance in the environment writes:

(7) $$\frac{d\left(-Mq_{e}\cdot R_{e}\right)}{dz}=-\epsilon \cdot M\cdot q_{e}\cdot R_{e}+\delta \cdot M\cdot q_{s}\cdot R_{s}+f_{ev}\cdot c\cdot \phi_{e}\cdot R_{e}-\eta \cdot M\cdot \frac{\partial \left(q_{e}R_{e}\right)}{\partial z}$$

where *ϕ*_*e*_ = *R*_*ev*_/*R*_*e*_ and *R*_*ev*_ is the ratio of the precipitation evaporation flux.

#### 4.1.2 Other Simplifying Assumptions and Differential Equations

To simplify the equations, as in (Romps, 2014) we assume that *q*_*s*_ is an exponential function of altitude:

(8) $$q_{s}=q_{s}\left(z_{0}\right)\cdot e^{-\gamma \cdot \left(z-z_{0}\right)}$$

where *γ* is a lapse rate in *m* ^−1^ calculated as *d ln*(*q*_*s*_)/*dz*.

For isotopes, we assume that the *R*_*s*_ is a power function of *q*_*s*_, consistent with a Rayleigh distillation:

$$R_{s}=R_{s}\left(z_{0}\right){\left(q_{s}/q_{s0}\right)}^{a_{s}-1}$$

Coefficient *α*_*s*_ represents the steepness of the *q* − *δD*_*v*_ gradient in cloudy regions and remains to be estimated. As in Duan et al. (2018), *R*_*s*_ is thus an exponential function of altitude:

(9) $$R_{s}=R_{s}\left(z_{0}\right)\cdot e^{-\left(\alpha_{s}-1\right)\cdot \gamma \cdot \left(z-z_{0}\right)}$$

We set:

$$q_{e}=h\cdot q_{s}$$

$$R_{e}=H\cdot R_{s}$$

Combining Equation (5) with Equations (3) and (8), we get the following differential equation for *h*:

(10) $$\frac{\partial h}{\partial z}=h\cdot \gamma -\frac{\delta }{1-\eta }\left(1-h\right)-\frac{f_{ev}\cdot \mu \cdot \gamma }{1-\eta }$$

where *μ* = *c*/(*M* ⋅ *q*_*s*_ ⋅ *γ*) represents the ratio of actual condensation (*c*) relative to the condensation if the ascent was adiabatic (*M* ⋅ *q*_*s*_ ⋅ *γ*). Similarly, combining Equation (7) with Equations (5) and (9), we get the following differential equation for *H*:

(11) $$\frac{\partial H}{\partial z}=H\cdot \gamma \cdot \left(\alpha_{s}-1\right)-\frac{\delta }{h\cdot \left(1-\eta \right)}\cdot \left(1-H\right)-\frac{f_{ev}\cdot \mu \cdot \gamma }{h\cdot \left(1-\eta \right)}\cdot H\cdot \left(\phi_{e}-1\right)$$

Note that these equations are only valid as long as *η* < 1, which will be the case in all our simulations (section 3.1.4). We now have two equations with four unknowns: *h*, *H*, *μ*, and *α*_*s*_. The condensation efficiency *μ* can be deduced from Equation (4):

(12) $$\mu =1-\frac{\epsilon }{\gamma }\cdot \left(1-h\right)$$

This equation, similar to one in (Romps, 2014), reflects the fact that condensation efficiency decreases when entrainment *ϵ* increases and when the entrained air is drier. If *ϵ* = 0 or *h* = 1, then *μ* = 1.

Similarly, the *q* − *δD*_*v*_ steepness *α*_*s*_ in cloudy air can be deduced from Equation (6):

(13) $$\alpha_{s}-1=\mu \cdot \left(\alpha_{eq}-1\right)+\frac{\epsilon }{\gamma }\cdot h\cdot \left(1-H\right)$$

This equation tells us that two effects control the steepness of the *q* − *δD*_*v*_ gradient. First, there is a “dilution effect”: if dry air is entrained, then the condensation efficiency *μ* decreases. This reduces *α*_*s*_ compared to *α*_*eq*_, i.e., compared to what we would expect from Rayleigh distillation. Second, there is an “isotopic contrast effect”: if depleted water vapor is entrained (*H* < 1), then *α*_*s*_ becomes steeper. This is how a depleting effect of rain evaporation in the environment can translate into a larger steepness in both regions, and eventually more depleted SCL.

#### 4.1.3 Numerical Solutions

To get analytical solutions for *h* and *H* (Romps, 2014), and Duan et al. (2018) assume that $h\cdot \frac{\partial q_{s}}{\partial z}\gg q_{s}\cdot \frac{\partial h}{\partial z}$ and that $H\cdot \frac{\partial R_{s}}{\partial z}\gg R_{s}\cdot \frac{\partial H}{\partial z}$. This allows them to calculate *h* and *H* as the solutions of a simple linear equation and of a second‐order polynomial, respectively. However, there are two issues with these solutions. First, although these solutions behave reasonably for *h* (Romps, 2014), they become very noisy, unstable or unrealistic for *H* when values for *ϵ*, *δ*, and *f*_*ev*_ that are diagnosed from LES outputs. This is because a powerful positive feedback exists between *α*_*s*_ and *H*: as *H* decreases, more depleted vapor is entrained in updrafts which increases the steepness *α*_*s*_; in turn, the stronger steepness *α*_*s*_ makes the subsidence more efficient at depleting the environment, further decreasing *H*. Duan et al. (2018) circumvented this problem by assuming *ϵ* and *δ* that are uniform with altitude and equal to each other, but it is at the cost of artificially reducing freedom for the solutions. Second, our hypothesis is that rain evaporation near the melting level affects the isotopic profiles down to the SCL. We thus want each altitude to feel the memory of processes at higher altitudes. The term with $\frac{\partial H}{\partial z}$ is thus a key ingredient in our framework.

Therefore, we choose to numerically solve the differential Equations (10) and (11). We start from an altitude of 5 km with *h* = 0.8 and *H* − 1 = −10‰. We do not start above 5 km because entrainment is more difficult to diagnose above the melting level (Section 3.1.4). We integrate Equations (10) and (11) down to the SCL top around 500 m. The resulting *h* profile is a function of the profiles of five input parameters: *γ*, *ϵ*, *δ*, *f*_*ev*_, and *η*. The *H* profile is a function of seven input parameters: *γ*, *ϵ*, *δ*, *f*_*ev*_, *η*, *α*_*eq*_, and *ϕ*_*e*_. These input parameters are all diagnosed from the LES simulations as detailed below. In each LES level, the input parameters are assumed constant and Equations (10) and (11) are integrated within each layer over 50 sublayers.

#### 4.1.4 Diagnosed Input Parameters

**Figure 10:**
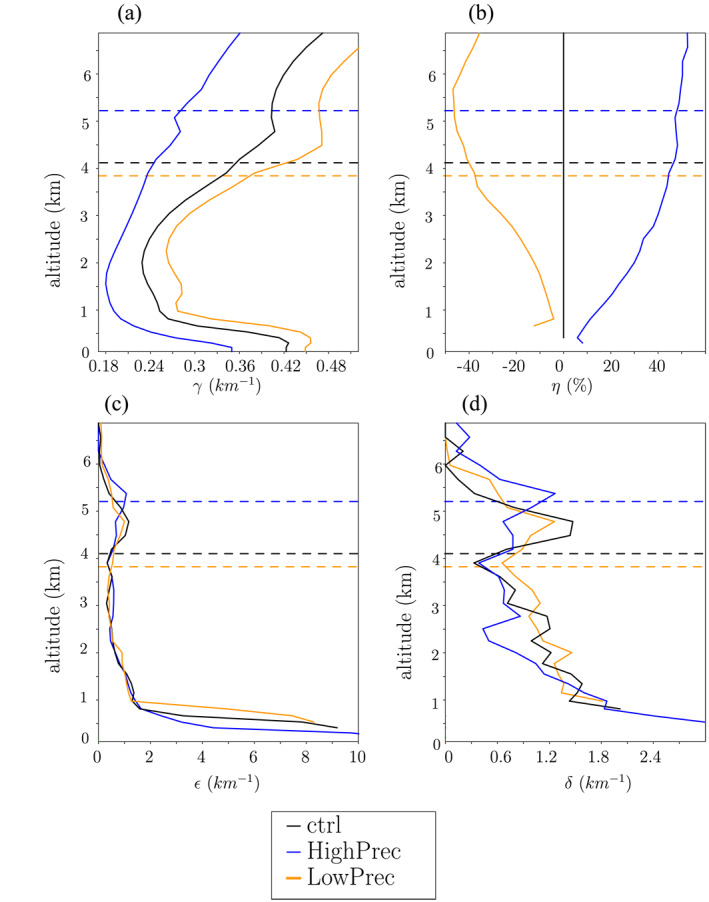
Input parameters for the simple model, for ctrl (black), HighPrec (blue), and LowPrec (orange). (a) Saturation specific humidity lapse rate *γ*; (b) ratio of large‐scale vertical mass flux over the cloudy mass flux; (c) entrainment rate; (d) detrainment rate.

Parameters *f*_*ev*_, *α*_*eq*_, and *ϕ*_*e*_ were already plotted in Figure 7 and discussed in section 2.6. Parameter *γ* is calculated from domain‐mean profiles. It is steeper in ctrl than in *ω* − 60 because of the steeper temperature gradient resulting from the drier air (Figure 10a). Parameter *η* = *M*_*LS*_/*M* is calculated from the net upward mass flux in cloudy regions *M* (Figure 10b), which is calculated as the average vertical velocity in cloudy regions multiplied by the area fraction of the cloudy region. Entrainment *ϵ* is diagnosed by using the conservation of the frozen moist static energy *m* (e.g., Hohenegger and Bretherton, 2011; Del Genio and Wu, 2010):

$$\frac{\partial m_{s}}{dz}=\epsilon \cdot \left(m_{e}-m_{s}\right)$$

where *m*_*s*_ and *m*_*e*_ are the frozen moist static energy in the cloudy region and the environment, respectively. The application of this equation is limited to the lower troposphere. Above the melting level, we would need to account for the precipitation of ice (Pauluis & Mrowiec, 2013) and for the lofting of rain. Therefore, we arbitrarily set a minimum of *ϵ* = 0.5 km^−1^ above the melting level. Entrainment is maximal in the subcloud layer, and decreases exponentially with height (Figure 10c), consistent with previous studies (De Rooy et al., 2013; Del Genio & Wu, 2010).

Finally, detrainment *δ* is deduced from *ϵ* and *M* using Equation (3). Detrainment shows the typical trimodal distribution (Johnson et al., 1999) (Figure 10d), with a first maximum just above the SCL top corresponding to the detrainment of shallow convection, a second maximum near the melting level corresponding to the detrainment of congestus convection, and a third maximum in the upper troposphere corresponding to the deep convection (not shown in Figure 10d).

We emphasize that our two‐column model applies on the full domain. Input profiles thus represent bulk properties that may hide large horizontal disparities. For example, we expect a deep overturning circulation in high‐cloud parts of the domain and a shallow overtiring circulation in low‐cloud or clear‐sky parts of the domain, with very different detrainment and evaporation properties (Text S3). Yet, the relative humidity and *δD*_*v*_ profiles simulated by the LES are remarkably homogeneous between the different parts of the domain (Text S3). This may be due to the disorganized state of convection in our simulations. Isolated cumulonimbi develop randomly in the domain and decay within a few hours, so that each location of the domain regularly undergoes the influence of deep‐convective processes. This prevents the building of strong horizontal gradients between high‐cloud and low‐cloud or clear‐sky parts of the domain. As a consequence, in our simulations, both deep and shallow overturning circulations simultaneously act on the domain‐mean relative humidity and *δD*_*v*_ profiles. This justifies mixing them together in our two‐column framework. Our framework may thus not apply so well in case of organized convection, in which stronger humidity and isotopic horizontal variations are expected to build at the mesoscale.

#### 4.1.5 Closure in the Subcloud Layer

To calculate the full *δD* profiles, we need as initial condition the isotopic ratio in the SCL. With this aim, we use a simple version of the SCL model of (Risi et al., 2020). We assume that water enters the SCL through surface evaporation and through downdrafts at the SCL top, and exits the SCL through updrafts at the SCL top. We neglect large‐scale forcing and rain evaporation, since they have a small impact in the SCL (Risi et al., 2020). The air flux of updrafts equals that of downdrafts. We define *r*_*u*_ = *q*_*u*_/*q*_1_ and *r*_*d*_ = *q*_*d*_/*q*_1_, where *q*_1_ is the mixing ratio in the SCL and *q*_*u*_ and *q*_*d*_ are the mixing ratios in updrafts and downdrafts at the SCL top. We assume that the water vapor is more enriched as the air is moister, following a logarithmic function: $R_{u}=R_{1}\cdot r_{u}^{\alpha_{u}-1}$ and $R_{d}=R_{1}\cdot r_{d}^{\alpha_{d}-1}$ where *R*_*u*_ and *R*_*d*_ are isotopic ratios in updrafts and downdrafts, and *α*_*u*_ and *α*_*d*_ are the *q* − *δD*_*v*_ steepness coefficients for updrafts and downdrafts. Water and isotopic budgets yield:

(14) $$R_{1}=\frac{R_{oce}/\alpha_{eq}\left(SST\right)}{h_{1}+\alpha_{K}\cdot \left(1-h_{1}\right)\cdot \frac{r_{u}^{\alpha_{u}}-r_{d}^{\alpha_{d}}}{r_{u}-r_{d}}}$$

where *R*_*oce*_ is the isotopic ratio at the ocean surface, *α*_*eq*_(*SST*) is the equilibrium fractionation coefficient at the sea surface temperature, *α*_*K*_ is kinetic fractionation coefficient (Merlivat & Jouzel, 1979) and *h*_1_ is the relative humidity normalized at the SST and accounting for ocean salinity: $h_{1}=q_{1}/q_{sat}^{surf}\left(SST\right)$, $q_{sat}^{surf}\left(SST\right)=0.98\cdot q_{sat}\left(SST\right)$ and *q*_*sat*_ is the humidity saturation as a function of temperature at the sea level pressure. We assume *δD*_*oce*_ = 0‰ and *h*_1_ is diagnosed from the LES.

For *r*_*u*_ and *r*_*d*_, we use values for the ctrl simulation, because small changes in *r*_*u*_ and *r*_*d*_ across simulations have only a marginal impact on *R*_1_ (Risi et al., 2020). Following Risi et al. (2020), we set *r*_*u*_ − 1 = 1.44% and *r*_*d*_ − 1 = −0.38%. For *α*_*u*_ and *α*_*d*_, Risi et al. (2020) had shown that they scale with *α*_*z*_ values above the SCL top, but with larger values especially for simulations with large‐scale ascent. We use an empirically fitting function: $\alpha_{u}=\alpha_{d}=1+100\cdot {\left(\tilde{\alpha_{z}}-1\right)}^{3}$, where $\tilde{\alpha_{z}}=1+\frac{ln\left(R\left(z_{SCT}\right)/R\left(z_{SCT}+1\;km\right)\right)}{ln\left(q\left(z_{SCT}\right)/d\left(z_{SCT}+1\;km\right)\right)}$ and *z*_*SCT*_ is the altitude of SCL top. Note that *α*_*u*_ and *α*_*d*_ are expected to scale with *α*_*z*_ only in case of disorganized convection (Text S4). In case of organized convection, strong horizontal gradients in *q* and *δD*_*v*_ are expected to build and the present closure would probably fail.

Finally, since the updraft region covers only a very small fraction of the domain, we assume that *R*_*e*_(*z*_*SCT*_) ≃ *R*_1_.

The procedure to calculate the full *δD*_*v*_ profiles is as follows:

1. Vertical profiles for *h*, *H*, and *α*_*s*_ are calculated through a downward integration of Equations (10)–(13) following Section 3.1.3
2. The vertical profile for a normalized version of *R*_*s*_, *R*_*s*,*norm*_ that satisfies *R*_*s*,*norm*_(*z*_*SCT*_) = 1, is calculated based on the *α*_*s*_ profile through an upward integration
3. The vertical profile for a normalized version of *R*_*e*_, *R*_*e*,*norm*_, is calculated as *R*_*e*,*norm*_ = *R*_*s*,*norm*_ ⋅ *H*.
4. From the *R*_*e*,*norm*_ profile, $\tilde{\alpha_{z}}$ is estimated
5. From *h*_1_ and $\tilde{\alpha_{z}}$, *R*_1_ is estimated
6. The full *R*_*e*_ profile can finally be calculated so that *R*_*e*_(*z*_*SCT*_) ≃ *R*_1_: *R*_*e*_ = *R*_*e*,*norm*_ ⋅ *R*_1_/*H*(*z*_*SCT*_)

#### 4.1.6 Evaluation of the Two‐Column Model

**Figure 11:**
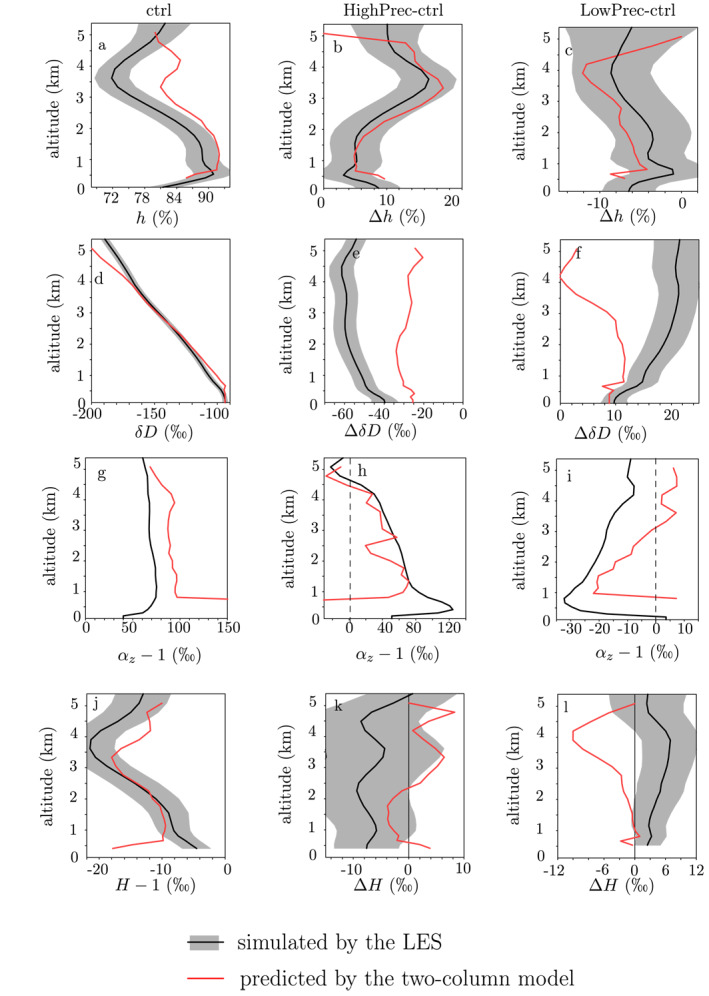
(a) Relative humidity *h* simulated by the LES (black) and predicted by the two‐column model (red) for the ctrl simulation. (b) Same as (a) but for the difference between HighPrec and ctrl. (c) Same as (b) but for the difference between LowPrec and ctrl. (d)–(f) Same as (a)–(c) but for the water vapor *δD*. (g)–(i) Same as (a)–(c) but for the steepness *α*_*z*_. (j–l) Same as (a)–(c) but for the relative enrichment of the environment relative to the updrafts (h) Error bars for the simulated profiles represent the spatial standard deviation (it was not plotted in g–i because the local steepness is very noisy). LES, large‐eddy simulations.

The two‐column model successfully captures the order of magnitude and the shape of the vertical profile of *h* for the ctrl simulation (Figures 11a), as well as the moister troposphere in HighPrec and the drier troposphere in LowPrec (Figures 11b and 11c). It successfully captures the vertical profile of *δD*_*v*_ (Figures 11b). It also captures the order of magnitude of the steepness *α*_*z*_ (Figures 11g), the sign of the *δD*_*v*_ and *α*_*z*_ differences between in HighPrec and ctrl (Figures 11e and 11h) and LowPrec and ctrl (Figures 11f and 11i).

However, the two‐column model underestimates the tropospheric depletion from ctrl to HighPrec by about half (Figures 11e) and the tropospheric enrichment from ctrl to LowPrec, especially in the middle troposphere (Figures 11f). These mismatches are caused by mismatches in the estimate of the relative enrichment of the environment relative to the cloudy region *H*. Although it is reasonably well predicted for the ctrl simulation (Figures 11j), the model fails to simulate the smaller *H* for HighPrec in the middle troposphere and the larger *H* for LowPrec almost everywhere. The two‐column model overestimates the impact of *η* and predicts a behavior for *H* that is too similar to that of *h*. It also underestimates the impact of rain evaporation (section 3.2.3). We could not find the exact reason for this shortcoming, but we acknowledge that the two‐column model hides many horizontal heterogeneity. We will have to keep this shortcoming in mind when interpreting the results.

### 4.2 Decomposition of Relative Humidity and *δD*_*v*_ Variations

To estimate the impact of the different input parameters on the *h* and *δD*_*v*_ profiles, we modify them one by one from the ctrl simulation to the HighPrec and from the ctrl simulation to LowPrec simulations.

#### 4.2.1 Decomposition of Relative Humidity

**Figure 12:**
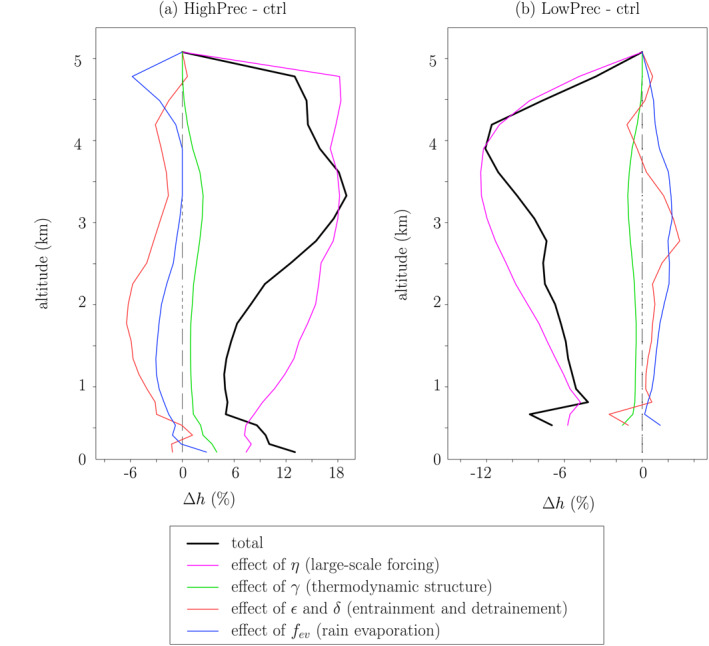
(a) Relative humidity difference between HighPrec and ctrl predicted by the two‐column model (black) and its contributions from variations of input parameters one by one: *η* (pink), *γ* (green), *ϵ* and *δ* (red), and *f*_*ev*_ (blue). (b) Same as (a) but for the difference between LowPrec and ctrl.

The moister troposphere in HighPrec is mainly due to the larger *η*, i.e., the direct moistening effect of large‐scale ascent (Figure 12a). The thermodynamic structure, entrainment, detrainment, and rain evaporation have a much smaller effect. Similarly, the drier troposphere in LowPrec is mainly due to the more negative *η*, i.e., the direct drying effect of large‐scale descent (Figure 12b).

Note that the direct effect of *η* on *h* in the environment may be overestimated in our simulations by prescribing a large‐scale vertical velocity profile that is horizontally uniform (Bao et al., 2017).

#### 4.2.2 Dilution Effect on *δD*_*v*_

**Figure 13:**
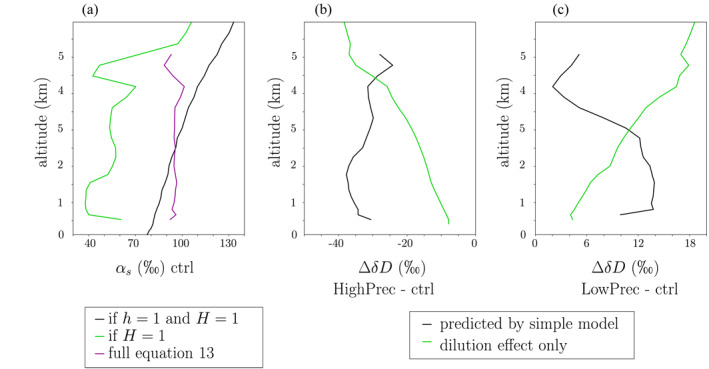
(a) Fractionation coefficient *α*_*eq*_ (black), corresponding to the steepness in the cloudy column *α*_*s*_ if *h* = 1 and *H* = 1; steepness *α*_*s*_ predicted if *h* < 1 and *H* = 1 (*α*_*s*_ = 1 + *μ* ⋅ (*α*_*eq*_ − 1)) (green); steepness *α*_*s*_ from the full Equation (13) (purple). (b) Difference in *δD*_*v*_ from ctrl to HighPrec predicted by the two‐column model (black) and predicted if accounting only for the dilution effect (green). (c) Same as (b) but for LowPrec.

A first effect impacting *δD*_*v*_ profiles is the dilution by entrainment (section 3.1.2). In the absence of entrainment (*ϵ* = 0), the steepness in the updraft column would be *α*_*s*_ = *α*_*eq*_ (Figure 13a, black). Because dry air is entrained, the condensation rate is reduced by the factor *μ* following Equation (12). According to Equation (13), this reduces the steepness (Figure 13a, green). This effect of entrainment can be understood as a mixing process: as the air rises and condensation proceeds, the remaining air is mixed with dry air from entrainment and with droplets that evaporate. Consistent with the concave‐down shape of the mixing lines, this leads to a reduction of the *q* − *δD*_*v*_ steepness (Figure 1, orange and cyan).

As a consequence of this “dilution effect,” tropospheric *δD*_*v*_ is less depleted than predicted by Rayleigh distillation. Since the troposphere is moister in HighPrec, entrained air leads to less evaporation of cloud droplets than in ctrl. This weaker “dilution effect” contributes to more depleted *δD*_*v*_ in HighPrec (Figure 13b, green). Reciprocally, since the troposphere is drier in LowPrec, the stronger “dilution effect” contributes to the more enriched *δD*_*v*_ in LowPrec (Figure 13c, green). Quantitatively, the contribution of this dilution effect on the SCL *δD*_*v*_ difference is 29% for HighPrec and 47% for LowPrec (Table 1). The contribution increases with altitude.

**Table 1:** *Difference of* *δD*_*v*_ *in the SCL Between HighPrec and ctrl and Between LowPrec and ctrl Simulated by the LES and Predicted by the Two‐Column Model, and the Contribution of the Dilution Effect*

Difference in SCL *δD*_*v*_ from ctrl	HighPrec	LowPrec
Total simulated by the LES (‰)	−40	10
Total predicted by the two‐column model (‰)	−30	11
Dilution effect (‰, %)	−9 (29%)	5 (47%)

Note that the two‐column model likely overestimates this contribution, because of the shortcoming mentioned in section 3.1.6. The fact that only one third of the *δD*_*v*_ difference remains when postcondensation effects are turned off (section 2.4) confirms that these contributions are overestimated.

### 4.3 Decomposition of *δD*_*v*_

**Figure 14:**
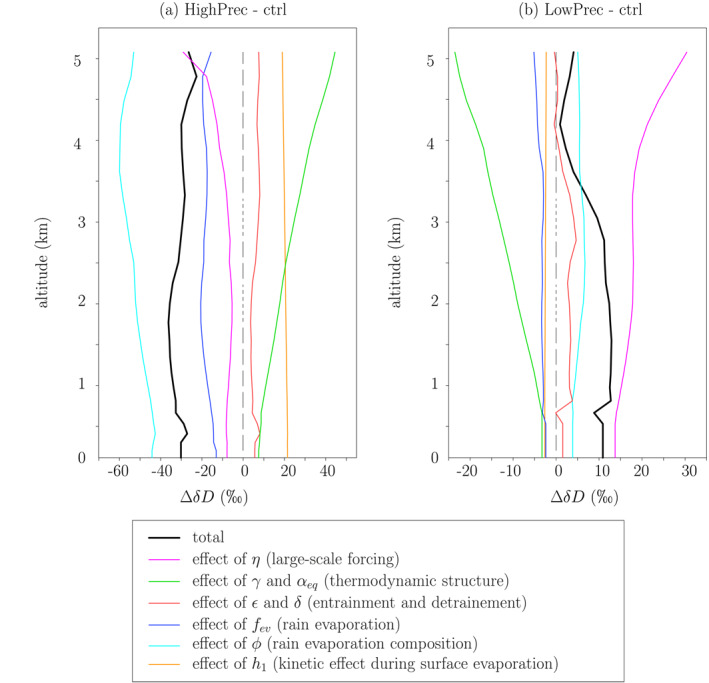
(a) *δD*_*v*_ difference between HighPrec and ctrl predicted by the two‐column model (black) and its contributions from variations of input parameters one by one: *η* (pink), *γ* and *α*_*eq*_ (green), *ϵ* and *δ* (red), *f*_*ev*_ (blue), *ϕ* (cyan), and *h*_1_ (orange). (b) Same as (a) but for the difference between LowPrec and ctrl.

In HighPrec, the more depleted troposphere is driven primarily by the effect of the smaller *ϕ*, i.e., the more depleted rain evaporation (Figure 14a, cyan). It explains 147% of the *δD*_*v*_ difference in the SCL (Table 2). The smaller rain evaporated fraction (smaller *f*_*ev*_) is the second main contributor (Figure 14a, blue, 43% in the SCL). This positive contribution is explained by the fact that evaporation has an overall enriching effect. The third main contributor is the larger *η* (i.e., large‐scale ascent), contributing to 26% of the *δD*_*v*_ difference. This contribution corresponds mainly to the “dilution effect” explained in section 3.2.2. The sum of these contributions exceeds 100%, because there are some dampening effects, especially *h*_1_: the moister surface relative humidity reduces the kinetic fractionation during surface evaporation.

**Table 2:** *Difference of* *δD*_*v*_ *in the SCL Between HighPrec and ctrl and Between LowPrec and ctrl Simulated by the LES and Predicted by the Two‐Column Model, and the Contribution of Different Effects*

SCL *δD*_*v*_ difference from ctrl	HighPrec	LowPrec
Total simulated by the LES (‰)	−40	10
Total predicted by the two‐column model (‰)	−30	11
Effect of *γ* and *α*_*eq*_ (‰, %)	8 (−25%)	−3 (−30%)
Effect of *ϵ* and *δ* (‰, %)	6 (−19%)	2 (14%)
Effect of *η* (‰, %)	−8 (26%)	14 (126%)
Effect of *f*_*ev*_ (‰, %)	−13 (43%)	−2 (−22%)
Effect of *ϕ*_*e*_ (‰, %)	−44 (147%)	4 (36%)
Including *ϕ*_*e*_ above 3 km (‰, %)	−23 (76%)	2 (23%)
Effect of *h*_1_ (‰, %)	22 (−72%)	−3 (−24%)

The sum of all the different effects, except the line “Including ϕ above 3 km,” is 100% of the predicted δDv difference. The line “Including ϕ above 3 km” is a part of “Effect of ϕ.”

In LowPrec, *η* becomes the main contribution to the *δD*_*v*_ difference in the SCL (126%), through the dilution effect (Figure 14b, pink, Table 2). The effect of the larger *ϕ*_*e*_, i.e., the more enriched rain evaporation, contributes to 36% to the *δD*_*v*_ difference in the SCL.

Compared to relative humidity, the relative contributions of the different processes to *δD*_*v*_ variations are remarkably uniform in the vertical. For example, in the SCL, half of the contribution of *ϕ* comes from *ϕ* above 3 km. This shows the strong “memory” of water vapor *δD*, which integrates processes downwards in the environment column, and then upward in the cloudy column. As a consequence, while considerations at a given altitude are relevant to understand the relative humidity (Romps, 2014), consideration of the full vertical profiles are necessary to understand the water vapor isotopic composition.

We recall that about one third of the *δD*_*v*_ difference from ctrl to HighPrec remains when the fractionation during condensate evaporation is deactivated. This remaining difference is associated with (1) the dilution effect, and (2) the portion of the *ϕ*_*e*_ contribution that is due to the more depleted rain due to more snow melt. The fact that the sum of these two contributions exceeds one third suggests that the two‐column model underestimates the effect of rain evaporation. This probably contributes to its underestimate of *δD*_*v*_ variations (Figures 11e and 11f)

## 5 Conclusion

### 5.1 Summary

**Figure 15:**
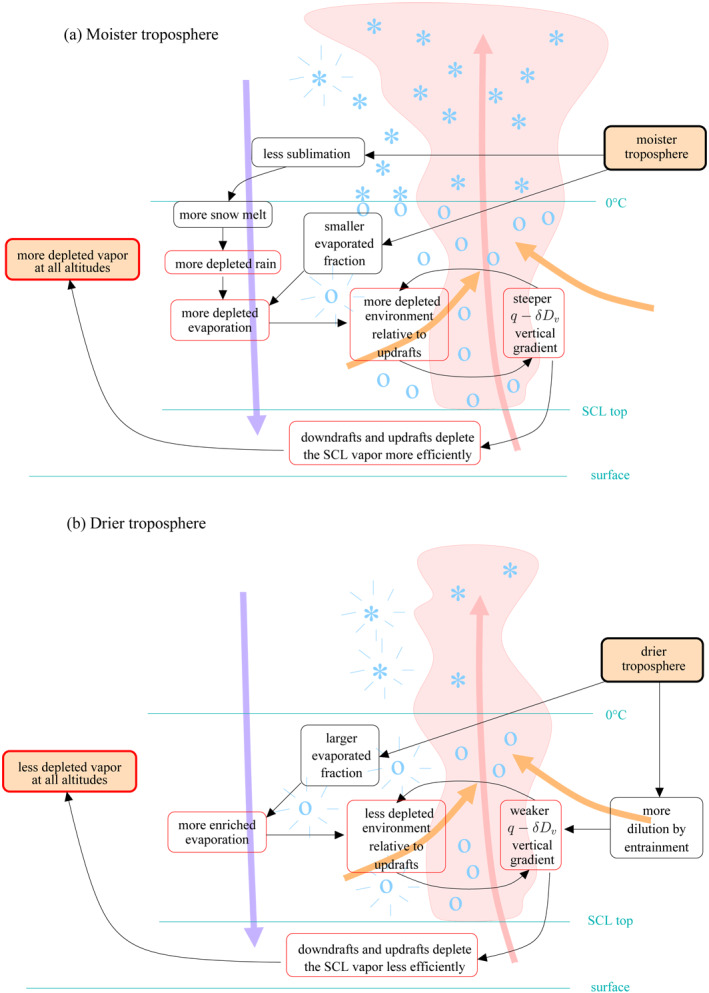
Schematic summarizing how a moister troposphere leads to more depleted vapor in the troposphere (a), and how a drier troposphere leads to more enriched water vapor (b). The black and red boxes represent standard water processes and isotopic processes, respectively. Blue stars indicate ice crystals or snow and blue circles indicate cloud droplets or rain. Blue rays indicate snow sublimation or rain evaporation. The pink and purple arrows, respectively, indicate the mean ascent in cloudy regions and mean descent in the environment.

The amount effect, i.e., the observed decrease in precipitation *δD* as precipitation rate increases, is the most salient feature in monthly mean isotopic observations over tropical oceans (Dansgaard, 1964). We confirm here that it is intimately related to the “vapor amount effect,” i.e., the observed decrease in water vapor *δD* as precipitation rate increases (Worden et al., 2007). This study gives a comprehensive and quantitative understanding of the processes underlying the “vapor amount effect,” at least in our LES simulations of disorganized convection (Figure 15).

We find that the relative humidity variations are essential to the “vapor amount effect,” with a triple effect on (1) the sublimation of snow aloft, (2) the fraction of rain that evaporates, and (3) the dilution of cloudy air by entrainment. Specifically, when the troposphere is moister (in terms of relative humidity), less snow sublimates and thus more snow is available for melting (Figure 15a). Snow melt results in rain that is more depleted relative to a liquid in equilibrium with the vapor, which leads to more depleted rain evaporation flux. When the troposphere is moister, the rain evaporated fraction is also smaller, making the rain evaporation flux even more depleted. The more depleted evaporation depletes the environment more efficiently relative to clouds. A positive feedback between the relative depletion of the environment and the steeper *q* − *δD*_*v*_ vertical gradient, involving cloud entrainment, allows to propagate the isotopic anomalies associated with rain evaporation downwards. The steeper *q* − *δD*_*v*_ gradient in the lower troposphere makes updrafts and downdrafts at the SCL top more efficient in depleting the SCL water vapor (Risi et al., 2020). Finally, since the more depleted SCL vapor serves as the initial condition for the full *δD*_*v*_ vertical profiles, the water vapor is more depleted at all altitudes in the troposphere (Figure 15a).

When the troposphere is drier, the reverse applies, but snow melt plays a smaller role and the *q* − *δD*_*v*_ vertical gradient is further weakened by the dilution of cloudy air by the entrainment of drier air, reducing the condensation efficiency (Figure 15b).

Coming back to our initial hypotheses to explain the “vapor amount effect,” the dominant role of rain evaporation and rain‐vapor diffusive exchanges confirms Hypothesis 3 (Lawrence et al., 2004; Lee & Fung, 2008; Risi, Bony, & Vimeux, 2008). For drier conditions, the role of entrainment in diluting cloudy air is reminiscent of Hypothesis 4.

The fact that the “vapor amount effect” is mediated by the tropospheric relative humidity probably explains why the amount effect can be observed only when the precipitation increase is associated with a change in the large‐scale circulation (Bailey et al., 2017; Bony et al., 2008; Moore et al., 2014; Risi et al., 2020). While the tropospheric relative humidity is very sensitive to the large‐scale circulation, it is almost invariant with sea surface temperature (Romps, 2014). For example, if precipitation increases because sea surface temperature increases without any change in large‐scale circulation, then the tropospheric humidity would remain almost constant (Romps, 2014), so the above‐mentioned mechanism cannot take place and there is no amount effect. In addition, the effect of relative humidity on isotopic profiles may be at play whatever the reason for the relative humidity variations, e.g., isentropic transport or shallow overturning circulations, or synoptic systems (Pierrehumbert, 1998; Zhang et al., 2004). However, in the latter case, horizontal advection may complicate the analysis (Noone et al., 2011).

## 6 Discussion and Perspectives

This study has investigated processes controlling isotopic profiles in idealized conditions. In particular, large‐scale horizontal gradients in humidity and *δD*_*v*_ were neglected. In reality, these gradients are expected to dampen the humidity and *δD* variations as a function of large‐scale vertical velocity (Risi et al., 2019).

In addition, our simulations depict disorganized convection with isolated, short‐lived cumulonimbi. In reality, convection may exhibit a wide range of convection organization degrees and types (Houze & Betts, 1981; Tobin et al., 2012), including mesoscale convective systems (Houze, 2004). Convective organization may alter our results in two ways. First, in case of organized and persistent convective systems, larger horizontal variations in humidity and *δD*_*v*_ are expected to build at the mesoscale, due to the reduced mixing between convective and low‐cloud or clear‐sky parts of the domain (Bretherton et al., 2005). The observation of strongly depleted water vapor in tropical cyclones (Lawrence et al., 2004), squall lines (Tremoy et al., 2014), and mature mesoscale convective systems in general (Kurita, 2013) support our expectation that larger horizontal variations are expected in case of organized convection. This violates our assumption that horizontal variations in humidity and *δD*_*v*_ profiles are small, necessary in our two‐column model and in our subcloud layer budget closure. Second, this paper highlights the important role of snow melt and rain evaporation in depleting the water vapor in case of large‐scale ascent. These processes are expected to be even stronger in stratiform regions of mature mesoscale systems, where all the rain arises from the widespread melting of snow near the melting level, and where the rain evaporation is boosted by the mesoscale downdraft that dries the lower troposphere (Houze, 1977, 2004). This may explain why observations show that stratiform regions are often more depleted than convective regions in squall lines (Risi, Bony, Vimeux, Chong, & Descroix, 2010; Tremoy et al., 2014), and why the water vapor is more depleted where the fraction of stratiform clouds is larger (Aggarwal et al., 2016; Kurita, 2013; Sengupta et al., 2020). Therefore, in our next study we will investigate water vapor isotopic profiles in LES with different convective organizations, such as squall lines (Robe & Emanuel, 2001; Muller, 2013) or tropical cyclones (Khairoutdinov & Emanuel, 2013; Muller & Romps, 2018).

Finally, this study highlights the key role of both microphysical processes (evaporation, snow melt) and macrophysical processes (entrainment) in the amount effect. While entrainment is partly resolved by grid‐scale motions, LES models rely strongly on microphysical and subgrid‐scale turbulence parameterizations in representing these processes. What is the sensitivity of the amount effect to these parameterizations? These processes are even more crudely parameterized in general circulation models (GCMs). How do GCMs represent these processes? More generally, what would be the added value of adding isotopic diagnostics when routinely comparing single‐column versions of GCMs to LES simulations? This is yet another question that we plan to address in the future.

## Supporting information

Supporting Information S1

## Data Availability

Information on SAM can be found on this web page: http://rossby.msrc.sunysb.edu/~marat/SAM.html. All simulation outputs used in this article have been submitted to the PANGEA data repository: https://doi.pangaea.de/10.1594/PANGAEA.918620.
