# Supplementary material for: Rain Evaporation, Snow Melt, and Entrainment at the Heart of Water Vapor Isotopic Variations in the Tropical Troposphere, According to Large‐Eddy Simulations and a Two‐Column Model
Source: J Adv Model Earth Syst. 2021 Apr 8;13(4):e2020MS002381. doi: 10.1029/2020MS002381 (PMC8047889; doi:10.1029/2020MS002381)
Supplement: Supplementary file 1 — Supporting Information S1 [file JAME-13-e2020MS002381-s001.pdf]

Supporting Information for "Rain evaporation, snow melt and entrainment at the heart of water vapor isotopic variations in the tropical troposphere, according to large-eddy simulations and a two-column model"

Camille Risi [1], Caroline Muller [1], Peter Blossey [2]

[1]Laboratoire de Meteorologie Dynamique, IPSL, CNRS, Ecole Normale Superieure, Sorbonne Universite, PSL Research University, Paris, France

[2]Department of Atmospheric Sciences, University of Washington, Seattle, USA

Contents of this file

1. Figure S1: Snapshots of the large-eddy simulations

2. Text S1: Robustness of the results with respect to the definition for clouds and the environment

3. Text S2: Simple equation for rain evaporation

4. Text S3: How do the deep and shallow overturning circulations contribute to set the domain-mean humidity and isotopic profiles?

5. Text S4: How do the $q-\delta D_v$ steepness coefficients in updrafts and downdrafts relate to vertical profiles?

Introduction

This supporting information illustrates the convective organization of the large-eddy simulations (Figure S1), assesses the robustness of the results with respect to the definition for clouds and the environment (Text S1) derives the simple equation for rain evaporation (Text S2), documents how the deep and shallow overturning circulations contribute to set the domain-mean humidity and isotopic profiles ((Text S3) and derives an equation relating the $q - \delta D_v$ steepness coefficients in updrafts and downdrafts to vertical profiles (Text S4).

Figure S1: Snapshots of the large-eddy simulations

To assess the convective organization in our simulations, we plot snapshots of maps of the precipitation rate and of near-surface water vapor δD_v. We can see that convection is disagregated, with isolated cumulonimbi (figure S1).

Text S1: Robustness of the results with respect to the definition for clouds and the environment

In our simple two-column framework, we decide to separate cloudy regions from their environment based on a threshold on cloud water content (e.g. Thayer-Calder and Randall (2015)): we define parcels as "cloudy" when the cloud water content exceeds 10^{-6} g/kg. In the previous studies, alternative definitions have been based on vertical velocity (e.g. Hohenegger and Bretherton (2011)) and/or buoyancy (e.g. Siebesma and Cuijpers (1995)), or position in altitude-equivalent potential temperature diagrams (Pauluis & Mrowiec, 2013). We thus test here the robustness of our results to different definitions, by defining "very cloudy regions" with cloud water content larger than 10^{-3} g/kg, "cloudy updrafts" with cloud water content larger than 10^{-6} g/kg and ascending vertical velocity, "saturated drafts" with relative humidity larger than 99%, "nearly-saturated drafts" with relative

humidity larger than 95%, and (7) "moist static energy updrafts" including all parcels falling into bins of frozen moist static energy in which the vertical velocity is positive (Pauluis & Mrowiec, 2013).

"Cloudy updrafts" and "nearly-saturated regions" are the most and least restrictive definitions respectively (Figure S2a,f). In all definitions, the cloudy region fraction remains below 10% except in the free lower and middle troposphere. In stricter definitions, the cloudy regions are characterized by a larger vertical velocity (Figure S2b) and a larger cloud water content (not shown). The entrainment is not strongly sensitive to the definition in the free troposphere (Figure S2c).

The ratio of the isotopic ratio in the rain evaporation over that in the environment vapor ($\phi = R_{ev}/R_v$) is not very sensitive to the definition for the ctrl (Figure S2e), but its value near the melting level is quite sensitive (Figure S2g). In all definitions, we can see the negative anomaly near the melting level, but it is much more negative in the loosest definitions. This is because in stricter definitions, the non-fractionating evaporation of cloud water droplets takes place in the environment. Since droplet evaporation takes place in shells around convective updrafts, and does not directly affect the environment, we chose a loose definition for the "cloudy regions".

The ratio of the large-scale mass flux over the cloudy mass flux, η, for HighPrec is larger in loose definitions (Figure S2h). This is because the cloudy regions incorporates cloudy downdrafts that compensate for the upward mass flux in cloudy updrafts. This large η in the loose definition may contribute to the overestimate of the direct effect of large-scale forcing on δD by the two-column model, and ultimately to the underestimate of the "vapor amount effect".

Text S2: Simple equation for rain evaporation

The quick equilibration between the rain and vapor motivates us to use a simple equation in which some mass q_{l0} of rain, with isotopic ratio R_{l0}, partially evaporates and isotopically equilibrates with some mass q_{e0} of vapor (subscript e for environment), with isotopic ratio R_{e0}. After the evaporation and equilibration process, the masses of rain and vapor are noted q_l and q_v:

$$q_l = q_{l0} - q_{ev}$$

$$q_e = q_{e0} + q_{ev}$$

where q_{ev} is the mass of evaporated rain water. The corresponding isotopic budget writes:

$$R_l \cdot q_l = R_{l0} \cdot q_{l0} - R_{ev} \cdot q_{ev}$$

$$R_e \cdot q_e = R_{v0} \cdot q_{v0} + R_{ev} \cdot q_{ev}$$

where R_l, R_e and R_{ev} are isotopic ratios in the final rain, final vapor and evaporation flux. Isotopic equilibrium writes:

$$R_l = \alpha_{eq} \cdot R_e$$

where α_{eq} is the equilibrium fractionation coefficient.

We define:

$$f_{ev} = \frac{q_{ev}}{q_{l0}}$$

$$g = \frac{q_{l0}}{q_{e0}}$$

$$\lambda = \frac{R_{l0}}{R_{e0}}$$

$$\phi = \frac{R_{ev}}{R_{e0}}$$

Re-arranging these equations, we get:

$$\phi = \frac{\lambda \cdot (1 + f \cdot g) - (1 - f) \cdot \alpha}{f \cdot (g \cdot (1 - f) \cdot \alpha + 1 + f \cdot g)}$$

If the mass of rain is much greater than than of vapor, i.e. $g \gg 1$, the equation becomes:

$$\phi = \frac{\lambda}{1 + (1 - f_{ev}) \cdot (\alpha_{eq} - 1)}$$

Therefore, ϕ scales with λ. In addition, ϕ increases with f_{ev} from $\phi = \lambda/\alpha_{eq}$ for $f_{ev} = 0$ (first order approximation) to $\phi = \lambda$ pour $f_{ev} = 1$ (total evaporation).

Text S3: How do the deep and shallow overturning circulations contribute to set the domain-mean humidity and isotopic profiles?

In our two-column model, we calculate bulk profiles for our input parameters γ (saturation specific humidity lapse rate), η (ratio of the large-scale vertical velocity relative to the cloudy region velocity), f_{ev} (evaporated fraction) and ϕ_e (relative isotopic ratio of rain evaporation), ϵ (entrainment rate) and δ (detrainment rate). These bulk profiles hide large horizontal disparities. In particular, we expect a deep overturning circulation in high-cloud parts of the domain and a shallow overturning circulation in low-cloud or clear-sky parts of the domain. How do the deep and shallow overturning circulations contribute to set the domain-mean humidity and isotopic profiles?

To address this question, we repeated all our diagnostics for each simulations on two sub-domains: the high-cloud columns (columns for which the cloud condensate mixing ratio

exceeds 10^{-6}g/kg in at least one level between 7 and 11 km) and the low-cloud/clear-sky columns (remaining columns).

Figure S3 shows the results for the ctrl simulation. Results for the other simulations are very similar (not shown). Our partition of the domain into high-cloud and low-cloud sub-domains effectively separates the circulation into its deep and shallow components (Figure S3a-c). High-cloud columns, covering 31% of the domain, show deep ascent and descent peaking in the upper troposphere Figure S3b) and have a higher high cloud fraction (Figure S3d), corresponding mainly to anvil clouds. In contrast, low-cloud/clear-sky columns cover 69% of the domain, show ascent and descent in the lower troposphere and descent above (Figure S3c) and have a higher low cloud fraction (Figure S3d). As a consequence, the η and δ profiles are very different (Figure S3e,f). The profiles for f_{ev} and ϕ_e are also very different (Figure S3g-h). In particular, ϕ_e is negative in high-cloud columns, due to the evaporation of rain arising from the melting of snow formed in the anvil clouds. Therefore, if we applied our two-column model separately for the two sub-domains, we would get very different relative humidity and δD_v profiles.

Yet, the relative humidity, δD_v and α_z profiles simulated by the LES are remarkably similar between the two sub-domains (Figure S3i-k). This means that the relative humidity and δD_v profiles are quickly homogenized between the sub-domains. This is likely due to the disorganized state of convection in our simulations. Isolated cumulonimbi develop randomly in the domain and decay within a few hours, so that each location of the domain regularly undergo the influence of deep convective processes (figure S1). This prevents the building of strong horizontal gradients between high-cloud and low-cloud/clear-sky sub-domains.

As a consequence, in our simulations, both deep and shallow overturning circulations simultaneously act on the domain-mean relative humidity and δD_v profiles. This justifies mixing them together in our two-column framework.

In case of organized convection however, stronger humidity and isotopic horizontal variations are expected to build at the meso-scale. Thus, the two sub-domains may show more contrasted humidity and δD_v profiles. Our two-column framework applied on domain-mean profiles may thus not apply so well.

Text S4: How do the $q-\delta D_v$ steepness coefficients in updrafts and downdrafts relate to vertical profiles?

In our simple SCL water budget, the efficiency of updrafts and downdrafts to deplete the SCL is quantified by the $q-\delta D_v$ steepness coefficients in updrafts and downdrafts ,α_u and α_d (Risi et al., 2020). In our LES, α_u and α_d scale with the domain-mean $q-\delta D_v$ steepness in the vertical, α_z (Risi et al., 2020). Is this scaling a universal property for all convective conditions, or is it specific to some conditions that our LES satisfy? To address this question, we theoretically calculate α_u as a function of α_z, and highlight the underlying hypotheses. In brief, we will show that α_u and α_d scale with α_z only if the convection is disorganized.

1. q and R as a function of w

In each grid point, at a given altitude, we assume that the specific humidity q and water vapor isotopic ratio R are controlled by vertical advection, a source term and a sink term. The source term may be turbulent mixing, advection or condensate evaporation The sink can be either mixing or advection without fractionation or condensation:

$$\frac{\partial q}{\partial t} = -w \cdot \frac{\partial q}{\partial z} + S - p \cdot q$$

$$\frac{\partial(q\cdot R)}{\partial t} = -w\cdot\frac{\partial(q\cdot R)}{\partial z} + S\cdot R_S - p\cdot q\cdot R\cdot\alpha_p$$

where w is the vertical velocity, S is the source term, p is the sink rate, R_S is the effective isotopic ratio of the source and α_p is the effective isotopic fractionation coefficient of the sink (e.g. $\alpha_p = 1$ for mixing or advection and $\alpha_p = \alpha_{eq}$ for condensation)

For simplicity and consistent with the approximations in our two-column model, we assume:

$$q(z) = q(z_0)\cdot e^{-\gamma\cdot(z-z_0)}$$

$$R(z) = R(z_0)\left(\frac{q(z)}{q(z_0)}\right)^{\alpha_z-1} = R(z_0)\cdot e^{-\gamma\cdot(\alpha_z-1)\cdot(z-z_0)}$$

At permanent state, we find:

$$q = \frac{S}{p - w\cdot\gamma}$$

$$R = R_S\cdot\frac{p-w\cdot\gamma}{p\cdot\alpha_p - w\cdot\gamma\cdot\alpha_z}$$

If we assume that horizontal variations of q and R associated with w are small, we can linearize q and R as a function of w:

$$q = \frac{S}{p}\cdot\left(1+\frac{\gamma}{p}\cdot w\right)$$

$$R = \frac{R_S}{\alpha_p}\cdot\left(1 + w\cdot\frac{\gamma}{p\cdot\alpha_p}\cdot(\alpha_z-\alpha_p)\right)$$

We can check that q increases with w (Risi et al., 2020). In general, we expect $\alpha_z > \alpha_p$, and thus R also increases with w (Risi et al., 2020).

2. Domain-mean q and R: $\bar{q}$ and $\bar{R}$

If S, p and γ do not vary too much with w, then we have:

$$\bar{q} = \frac{S}{p} \cdot \left(1 + \frac{\gamma}{p} \cdot \bar{w}\right)$$

Since $\bar{w} = 0$, we have:

$$\bar{q} = \frac{S}{p}$$

If in addition, if α_p and α_z do not vary too much with w, then we have:

$$\bar{R} = \frac{R_S}{\alpha_p}$$

We can thus rewrite q and R as:

$$q = \bar{q} + \frac{S \cdot \gamma}{p^2} \cdot \bar{w} \tag{1}$$

$$R = \bar{R} \cdot \left(1 + w \cdot \frac{\gamma}{p \cdot \alpha_p} \cdot (\alpha_z - \alpha_p)\right)$$

3. Effective q and R in updrafts: q_u and R_u

Following (Risi et al., 2020), M_u, q_u and R_u are defined as:

$$M_u = \frac{1}{n} \cdot \sum_U \rho \cdot w$$

$$M_u(q_u - \bar{q}) = \frac{1}{n} \cdot \sum_U \rho \cdot w \cdot (q - \bar{q})$$

$$M_u(R_u \cdot q_u - \bar{q} \cdot \bar{R}) = \frac{1}{n} \cdot \sum_U \rho \cdot w \cdot (qR - \bar{q}\bar{R})$$

where U is the ensemble of grid points where $w > 0$ and n is the total number of grid points.

Thus,

$$q_u = \bar{q} + \frac{1}{n \cdot M_u} \cdot \sum_U \rho \cdot w \cdot (q - \bar{q})$$

Using equation 1 , we get:

$$q_u = \bar{q} \left(1 + \frac{\gamma}{p} \cdot \frac{\sum_U \rho \cdot w^2}{\sum_U \rho \cdot w} \right)$$

Let's define

$$\sigma = \frac{\sum_U \rho \cdot w^2}{\sum_U \rho \cdot w}$$

This corresponds to the ratio of w variance over the w mean among ascending grid points. We thus have:

$$q_u = \bar{q} \cdot \left(1 + \frac{\gamma}{p} \cdot \sigma \right) \tag{2}$$

Similarly,

$$R_u = \bar{R} \cdot \frac{1 + \frac{\gamma \cdot \alpha_z}{p \cdot \alpha_p} \sigma}{1 + \frac{\gamma}{p} \cdot \sigma} \tag{3}$$

4. $q - \delta D_v$ steepness coefficient in updrafts: α_u

α_u is defined as:

$$\alpha_u = 1 + \frac{ln(R_u/\bar{R})}{ln(q_u/\bar{q})}$$

Using equations 2 and 3, we get:

$$\alpha_u = 1 + \frac{ln\left(1 + \frac{\gamma \cdot \alpha_z}{p \cdot \alpha_p}\sigma\right) - ln\left(1 + \frac{\gamma}{p} \cdot \sigma\right)}{ln\left(1 + \frac{\gamma}{p} \cdot \sigma\right)}$$

Since we assumed that horizontal variations of q and R associated with w were small, we can linearize the logarithms:

$$\alpha_u = 1 + \frac{\frac{\gamma \cdot \alpha_z}{p \cdot \alpha_p}\sigma - \frac{\gamma}{p} \cdot \sigma}{\frac{\gamma}{p} \cdot \sigma} = \alpha_z/\alpha_p$$

Therefore, α_u scales with α_z, consistent with our LES simulations (Risi et al., 2020). Similarly, we can show that α_d scales with α_z. We expect α_u and α_d to equal α_z (if $\alpha_p = 1$) or to be slightly smaller (if $\alpha_p > 1$). The slightly larger values of α_u and α_d in our simulations may be due to slightly violated assumptions, or to the fact that we use α_z 1 km above the SCL top.

5. Discussion of the underlying hypotheses

We assumed two main hypotheses:

1. q and R variations associated with w are small
2. S, p, γ, α_p and α_z do not vary too much with w.

Together, these assumptions mean that q and R variations with w are small.

We can check that this is the case in our simulations: q and R anomalies relative to the domain-mean remain are smaller than 10% and 2% of the domain-mean respectively at the SCL top (Risi et al., 2020). This is because the simulated convection is disorganized: isolated cumulonimbi develop randomly in the domain and decay within a few hours, so that each location of the domain regularly undergo the influence of deep convective processes (figure S1). This is why in our LES simulations, α_u and α_d scale with α_z.

In contrast, in a case of organized convection, we expect q and R to vary much more strongly at the meso-scale. First, meso-scale convective systems live longer, so some regions of the domain can remain several days without undergoing any effect of deep convective processes. This may contribute to the drier troposphere observed when convection is more organized (Bretherton & Khairoutdinov, 2015; Tobin et al., 2012). Second, meso-scale convective systems are larger, reducing the effect of horizontal advection and mixing, and thus allowing strong horizontal gradients to build. In other words, meso-scale convective systems are better protected from the environment, and vice versa (Tobin et al., 2012). The observation of strongly depleted water vapor in tropical cyclones (Lawrence et al., 2004), squall lines (Tremoy et al., 2014) and mature meso-scale convective systems in general (Kurita, 2013) support our expectation that larger horizontal variations in R are expected in case of organized convection.

Therefore, in case of organized convection, S, p, γ, α_p and α_z may strongly vary with w. Consequently, α_u and α_d may not scale with α_z. Rather, horizontal variations in R at the meso-scale may control α_u and α_d.

References

Bretherton, C. S., & Khairoutdinov, M. F. (2015). Convective self-aggregation feedbacks in near-global cloud-resolving simulations of an aquaplanet. *Journal of Advances in Modeling Earth Systems*, *7*(4), 1765–1787.

Hohenegger, C., & Bretherton, C. S. (2011). Simulating deep convection with a shallow convection scheme. *Atmospheric Chemistry and Physics*, *11*, 10389–10406.

Kurita, N. (2013). Water isotopic variability in response to mesoscale convective system over the tropical ocean. *Journal of Geophysical Research*, *118(18)*, 10-376.

Lawrence, J. R., Gedzelman, S. D., Dexheimer, D., Cho, H.-K., Carrie, G. D., Gasparini, R., ... Biggerstaff, M. I. (2004, March). Stable isotopic composition of water vapor in the tropics. *J. Geophys. Res.*, *109*, D06115, doi:10.1029/2003JD004046. doi: 10.1029/2003JD004046

Pauluis, O. M., & Mrowiec, A. A. (2013). Isentropic analysis of convective motions. *Journal of the atmospheric sciences*, *70*(11), 3673–3688.

Risi, C., Muller, C., & N, B. P. (2020). What controls the water vapor isotopic composition near the surface of tropical oceans? results from an analytical model constrained by large-eddy simulations. *Journal of Advances in Modeling Earth Systems*.

Siebesma, A., & Cuijpers, J. (1995). Evaluation of parametric assumptions for shallow cumulus convection. *Journal of the atmospheric sciences*, *52*(6), 650–666.

Thayer-Calder, K., & Randall, D. (2015). A numerical investigation of boundary layer quasi-equilibrium. *Geophysical Research Letters*, *42*(2), 550–556.

Tobin, I., Bony, S., & Roca, R. (2012). Observational evidence for relationships between the degree of aggregation of deep convection, water vapor, surface fluxes and radiation. *Journal of Climate*.

Tremoy, G., Vimeux, F., Soumana, S., Souley, I., Risi, C., Cattani, O., ... Oi, M. (2014). Clustering mesoscale convective systems with laser-based water vapor delta18O monitoring in Niamey (Niger). *J. Geophys. Res.*, *119(9)*, 5079-5103, DOI: 10.1002/2013JD020968.

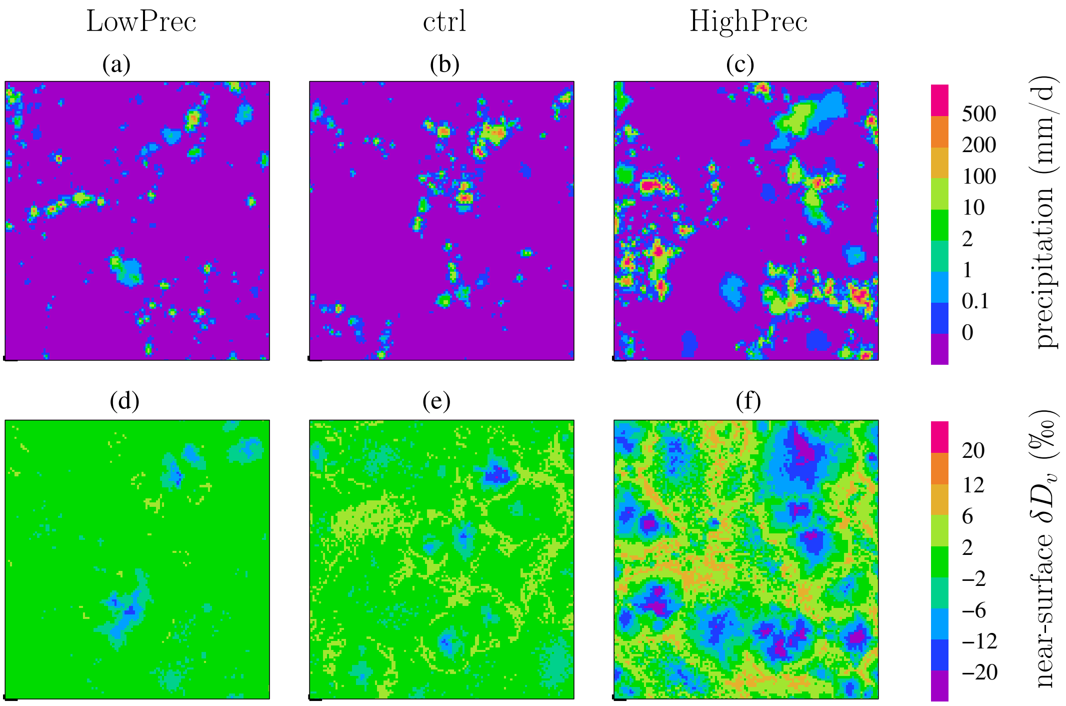

Figure S1. Snapshots of maps of the precipitation rate (a-c) and of near-surface water vapor δD_v (d-f) for LowPrec (a,d), ctrl (b,e) and HighPrec (c,f).

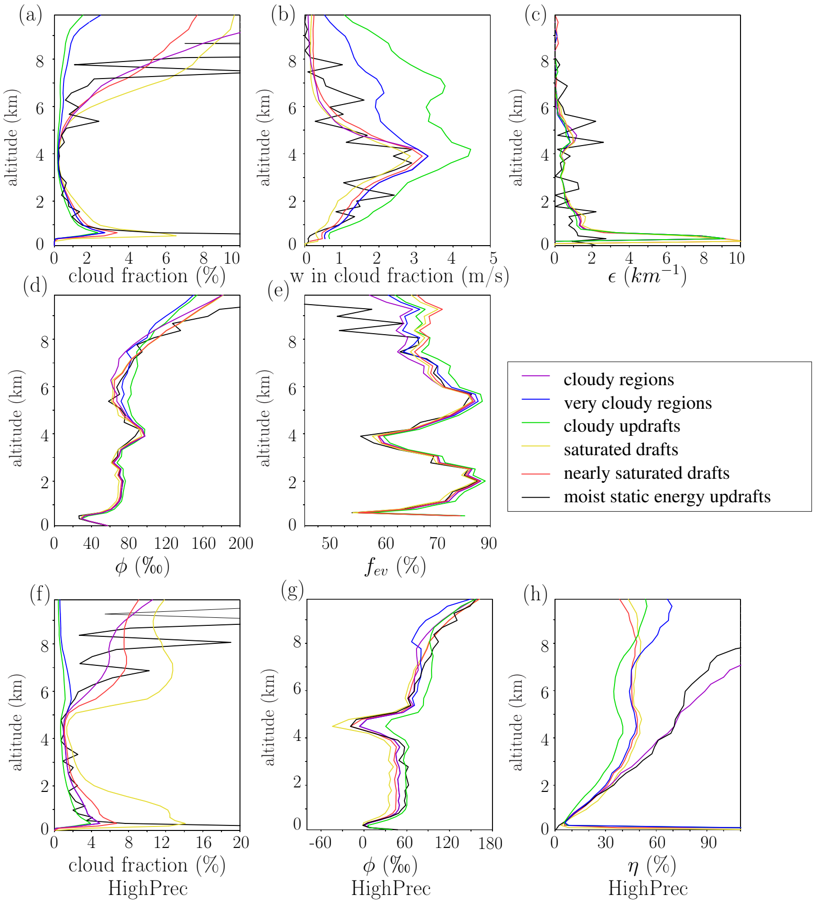

Figure S2. (a-e): Vertical profiles for the ctrl simulation. (a) fraction of the domain-area covered by cloudy regions. (b) Vertical velocity w in average over the cloudy regions. (c) Entrainment rate ϵ diagnosed from the frozen moist static energy budget as explained in the article. (d) $\phi = R_{ev}/R_e$, expressed in ‰; (e) rain evaporated fraction f_{ev}. The different colors show the different definitions for the cloudy regions: "cloudy regions" (purple), "very cloudy regions" (blue), "cloudy updrafts" (green), "saturated drafts" (yellow), "nearly saturated drafts" (red), and "moist static energy updrafts" (black). (f) Same as (a) but for HighPrec. (g) Same as (d) but for HighPrec. (h) Ratio of the large-scale mass flux over the cloudy mass flux, η, for HighPrec.

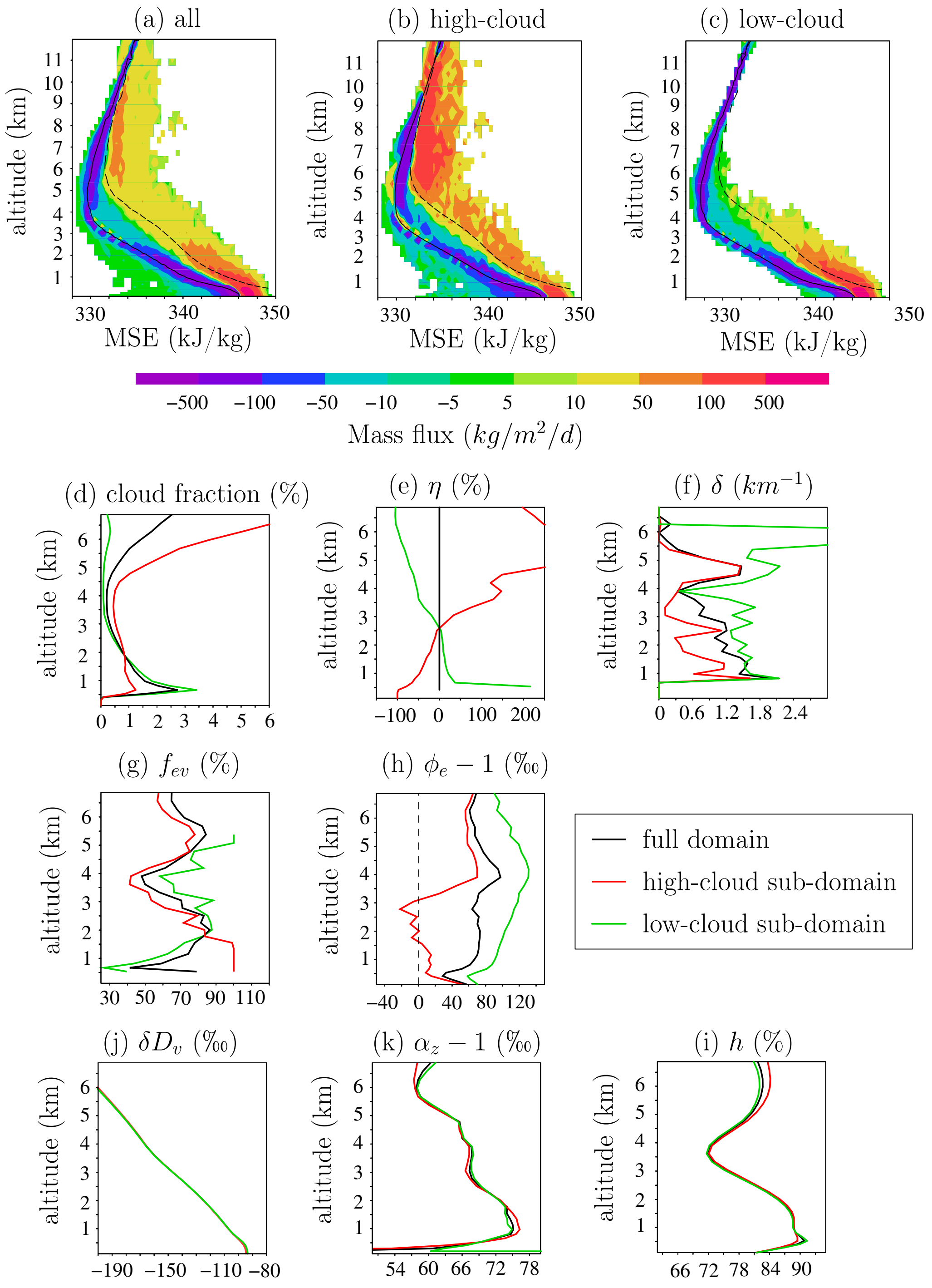

Figure S3. (a) Profiles of vertical mass flux (vertical velocity multiplied by the proportion of samples and density) binned as a function of moist static energy over the full domain, for the ctrl simulation. (b) Same as (a) but for the high-cloud sub-domain. (c) Same as (a) but for the ow-cloud/clear-sky sub-domain. (d) Cloud fraction for the full domain (black), high-cloud sub-domain (red) and low-cloud/clear-sky domain (green). (e-h) Same as (d) but for the η, δ, f_{ev} and ϕ_e profile. (i-k) Same as (d) but for the h, δD_v and α_z profiles. The high-cloud and low-cloud/clear-sky sub-domains cover respectively cover 31% and 69% of the domain in the ctrl simulation.
